# Perioperative, Functional, and Oncologic Outcomes of On-Clamp Versus Off-Clamp Partial Nephrectomy: An Updated Meta-analysis of 9027 Patients

**DOI:** 10.5152/tud.2023.22207

**Published:** 2023-03-01

**Authors:** Hosam Serag, Ayman Agag, Naufal Naushad, Ankur Mukherjee, Maria Harrington-Vogt, Abdalla Ali Deb

**Affiliations:** 1 Department of Urology, University Hospitals Birmingham, Birmingham, UK; 2 Department of Urology, Frimley Park Hospital, Camberley, UK; 3 Department of Urology, North Tees University Hospital, Stockton, UK; 4 Department of Urology, James Cook University Hospital, Middlesbrough, UK

**Keywords:** On-clamp, off-clamp, partial nephrectomy, meta-analysis

## Abstract

**Objective:**

The aim of this study was to determine the comparative efficacy and safety between on-clamp and off-clamp partial nephrectomy in patients with renal masses.

**Materials and methods:**

This systematic review was pre-registered on The International Prospective Register of Systematic Reviews (PROSPERO) (CRD42022339127). PubMed, Scopus, and Web of Science were searched. A manual search was also conducted to avoid missing relevant studies. All observational and experimental studies reporting the comparative efficacy and/or safety of on-clamp versus off-clamp partial nephrectomy were included. Outcomes were divided into 3 categories: perioperative, functional, and oncologic outcomes. Risk of bias was assessed using the The Risk Of Bias In Non-randomized Studies of Interventions (ROBINS-I) and revised Cochrane ROB-II tool for nonrandomized and randomized studies, respectively. Fixed- and random-effect models were implemented to pool the mean difference and log odds ratio of continuous and dichotomous outcomes, respectively. A leave-one-out sensitivity analysis was conducted to determine if the effect size was driven by a single study, and Egger’s regression test was used to assess publication bias.

**Results:**

Forty-two studies were meta-analyzed. The on-clamping method showed greater benefit when compared to the off-clamping technique in terms of perioperative (estimated blood loss and blood transfusion), functional (estimated glomerular filtration rate), and oncologic outcomes (tumor resection time). However, it is associated with higher risk for complications. Most studies were of moderate-to-serious risk of bias.

**Conclusion:**

On-clamping shows superiority in terms of estimated blood loss, blood transfusion, estimated glomerular filtration rate, and tumor resection time. However, it is associated with increased risk of complications. The selection of the technique should be tailored per individual case based on their comorbidities and preoperative risk profile.

**Main Points**

- The on-clamping method is associated with greater benefits than the off-clamping technique in terms of perioperative outcomes (i.e., estimated blood loss and postoperative blood transfusion).
- The on-clamping method is superior to the off-clamping technique regarding the postoperative estimated glomerular filtration rate and tumor transection time.
- Although the on-clamping method has shown superiority over the off-clamping method, it was reported to correlate with greater risk for postoperative overall complications, particularly acute kidney injury.
- The selection of the clamping method (on vs. off) should be determined and tailored according to each patient individually based on pre-interventional risk profile.
- Available evidence highlighted in this updated meta-analysis relies mainly on retrospective cohort studies. Therefore, more properly designed, long-term randomized controlled trials with large sample sizes are still warranted to confirm our observations.

## Introduction

Evidence indicates that the incidence of kidney cancer is remarkably increasing after the vast spread of imaging studies, which enabled early diagnosis and discovery of incidental findings suggestive of kidney cancer.^1^ In addition, estimates indicate that renal tumors are relatively common. However, the incidence of kidney cancer is not the highest among the general population, with an estimated worldwide incidence of 214 000 cases per year. In the same context, kidney cancer has been estimated to lead to 143 000 deaths annually, being the 16th leading cause of death globally.^2-6^ Managing renal tumors is variable, including active surveillance, ablation, or surgery. Therefore, it should be conducted according to a discussion between the physician and patient to elaborate on the risks and benefits of each approach.

According to the National Comprehensive Cancer Network, the American Urological Association, and the European Association of Urology (EAU), partial nephrectomy (PN) is recommended if anatomically possible.^7-9^ Moreover, it has been shown that PN is now considered the gold standard for managing resectable cT1 renal tumors.^7,9^ The oncological outcomes of PN are similar to radical nephrectomy, in addition to enhancing survival outcomes secondary to the partial preservation of renal functions.^1,10^ More recent investigations demonstrated that robot-assisted partial nephrectomy (RAPN) has favorable functional and perioperative outcomes for both laparoscopic and open modalities.^11-14^ Different factors have been proposed to determine postoperative renal functions, including volume of preserved renal tissue, preoperative renal functions, and warm ischemia time (WIT). Studies showed that WIT is a major factor that can be modified to enhance outcomes.^15,16^

The off-clamp or zero-ischemia technique has been recently proposed to reduce WIT and enhance the efficacy and safety of RAPN compared to the on-clamping approach, particularly in preserving postoperative renal functions.^17-19^ In addition, various meta-analyses compare off-clamp and on-clamp PN.^20-22^ However, evidence from these studies regarding the superiority of either of these techniques remains controversial, in addition to not being comprehensive in including all the available studies in the literature. Therefore, we aimed to conduct the current study to provide the most updated and comprehensive evidence comparing the efficacy and safety of off-clamp and on-clamp PN techniques.

## Materials and Methods

This updated systematic review and meta-analysis was conducted as per the preferred reporting items for systematic reviews and meta-analyses (PRISMA) guidelines and pre-registered on PROSPERO (registration number: CRD42022339127). We employed the Population, Intervention, Comparison, Outcomes, and Study Design (PICOS) framework in designing our research: the population included patients with renal tumors undergoing PN; the intervention included the on-clamp technique; the control included the off-clamp technique; outcomes included perioperative, functional, and oncologic outcomes; and the study design included comparative observational (cohort) and interventional [randomized controlled trials (RCTs)] studies. Of note, we included any studies that compared the on-clamp to the off-clamp technique regardless of the surgical method itself (i.e., open PN, robotic PN, or laparoscopic PN).

### Definition of Outcomes and Inclusion Criteria

The following criteria were considered in the screening process to retrieve relevant studies for our intended outcomes: (1) studies that compared the efficacy and/or safety of off-clamp versus on-clamp PN, (2) studies that were original investigations, including cohort studies (whether prospective or retrospective), RCTs, case–control studies, and quasi-experiments, and (3) studies that included human participants only with renal tumors. On the other hand, studies that (1) were not original (like thesis, reviews, protocols, commentaries, abstract-only articles, and posters), (2) included non-human subjects (like in vitro and in vivo studies), and (3) did not compare any related outcomes to off-clamp versus on-clamp PN were excluded from the analysis and data synthesis. However, it should be noted that we considered relevant meta-analyses to be discussed and compared with our final results.

### Search Strategy

On May 15, 2022, 3 electronic databases were searched: PubMed, Scopus, and Web of Science using a combination of relevant keywords from relevant studies and previous reviews. Based on these words, we searched each database based on the search terms and conditions and adjusted the search terms accordingly. For instance, we used the following term: [(clamp OR clamping OR “off-clamp” OR Clampless OR “on-clamp” OR “Warm ischemia time” OR WIT OR “Zero ischemia”) AND (“partial nephrectomy” OR “nephron sparing surgery”) AND (“robot-assisted” OR “robotic-assisted” OR robot OR robotic OR laparoscopic OR RALPN OR RAPN)] for PubMed. These search criteria were then adjusted for other databases as per their guidelines.

Additionally, we conducted a manual search strategy to find any relevant article that might be missed when conducting the electronic search strategy. This strategy included 3 approaches: (1) reading the reference lists of included articles, (2) screening “similar articles” to included studies through PubMed, and (3) searching Google Scholar. Only the first 200 records from Google Scholar were screened as per published recommendations.^23^ We also searched the references of relevant reviews not to miss any potentially relevant investigation.

### Screening Strategy

After retrieving all relevant articles found by our search strategy, we grouped them into 1 EndNote library to detect and eliminate all potential duplicates among the different databases. Then, we extracted the remaining citations into an Excel sheet to facilitate the screening process. We grouped these citations by their titles, abstracts, authors, and journals where they were published, DOIs, and URLs. Moreover, each citation was given an ID to prevent overlapping and facilitate the identification of each article.

The screening strategy was conducted by at least 2 members against our inclusion and exclusion criteria. The first step was to screen articles by titles and abstracts, while the following step involved full-text screening. The decision of each reviewer was blinded from the other not to induce any bias in the selection process. Finally, a senior member compared the results of the screening and conducted a discussion on the differences. Disagreements were resolved in this discussion using the supervisor’s opinion whenever needed.

### Data Extraction

After reaching a final list of included articles, a senior member will thoroughly go through these articles to plan all extractable data to design a suitable extraction sheet. A pilot extraction task will validate the extraction sheet by some of the study members before being used to extract relevant data to test the validity of the sheet to retrieve all relevant outcomes. At least 2 members will extract each included article, and any conflict will be resolved with a group discussion.

The sheet was mainly designed to extract data that can be divided into 3 main parts, including the baseline characteristics part (which is designed to accommodate reference for each study, study design, country, year of publication, the definition of intervention and control cases, age and gender of participants per each group, renal/nephelometry score, Charlson Comorbidity Index, Eastern Cooperative Oncology Group (ECOG) Performance Status Scale, and follow-up duration), outcome part (including perioperative, functional, and oncologic outcomes), and quality assessment and risk of bias (ROB) part.

Perioperative outcomes included operative time, estimated blood loss (EBL), length of hospital stay (LoS), overall complications, conversion to open surgery, re-intervention, any major bleeding, acute kidney injury (AKI), and transfusion rate. Functional outcomes included postoperative estimated glomerular filtration rate (eGFR), postoperative hemoglobin (Hb), and creatinine. Meanwhile, oncological outcomes included tumor size, reconstruction time, and positive surgical margin.

### Quality Assessment

The quality of included studies was assessed using the Newcastle–Ottawa Scale (NOS) for nonrandomized studies and the revised Cochrane risk of bias (ROB2) tool in RCTs. We provided the manual of each tool for all members to obtain the best quality. Both data extraction and quality assessment were conducted by 2 reviewers, and any differences were solved by consulting the senior author.

### Statistical Analysis

All analyses were conducted using STATA Software (Version 17). Fixed- and random-effect models were chosen based on encountered heterogeneity, where the random-effect model was chosen if significant heterogeneity was found (measured by *I* ^2^ ≥ 50% or *P* < .05). When significant heterogeneity was observed, meta-regression based on sample size was conducted to determine if sample size was a significant contributor to heterogeneity.

For continuous outcomes, the restricted maximum likelihood method was used to pool the mean difference (MD) and its corresponding 95% CI when heterogeneity was observed (random-effect model); otherwise, the inverse-variance method was used (fixed-effect model). However, in dichotomous outcomes, the log odds ratio (logOR) and its corresponding 95% CI was pooled using the Mantel-Haenszel method.

Finally, a leave-one-out sensitivity analysis was conducted by excluding 1 study at a time to determine whether or not the reported effect estimate was driven by a single study. The risk of publication bias was assessed using Egger’s regression test and funnel plot, where the trim-and-fill method would be applied if significant bias was encountered. In meta-analyses of <10 studies, the assessment of risk of publication bias was not feasible.

## Results

### Search Results

**Figure 1.**
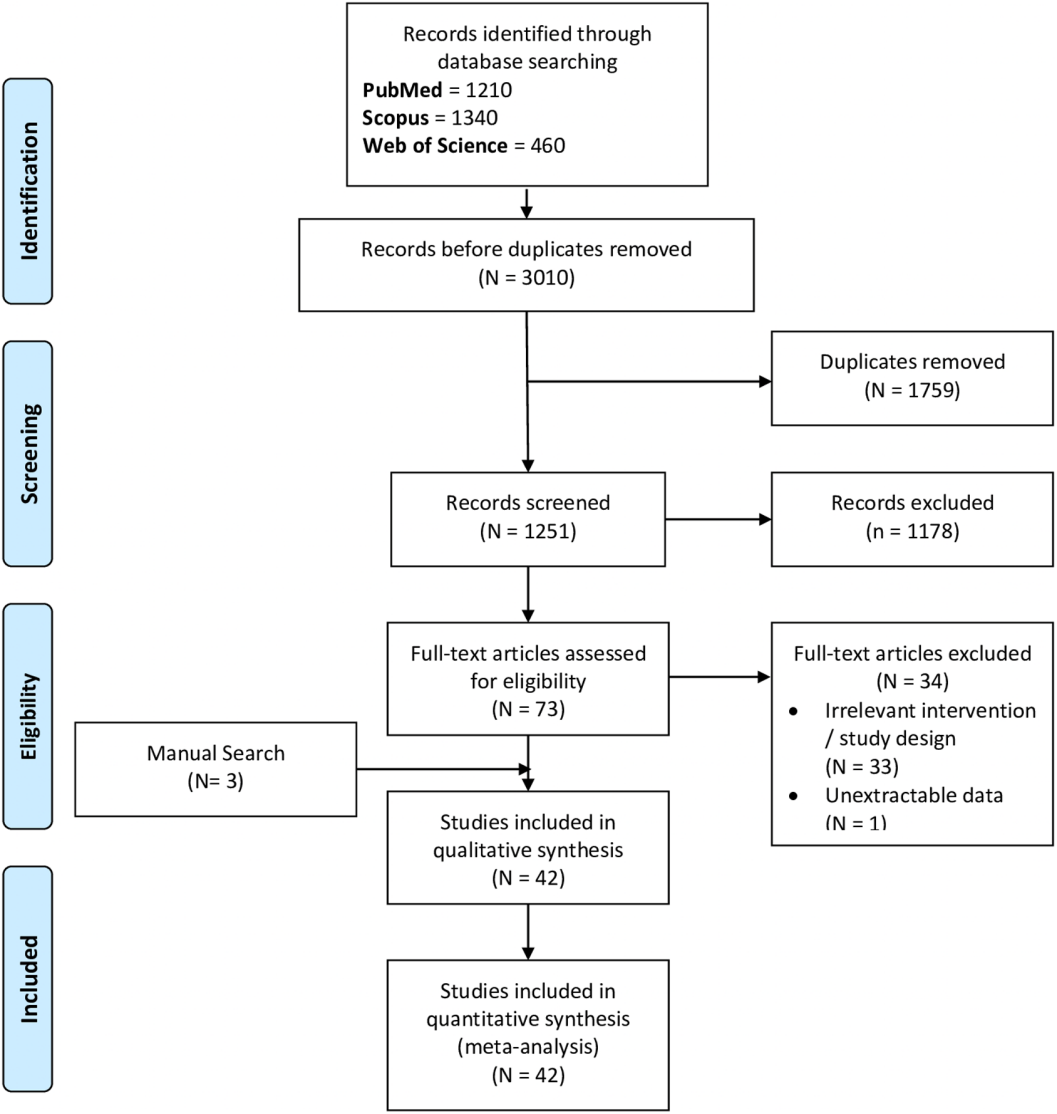
PRISMA flow diagram exhibiting the search process and inclusion of included studies.

The electronic search strategy yielded 3010 citations, exported into an EndNote library. We then removed 1759 citations identified as duplicates by the program, while the rest (n = 1251) were eligible for the title and abstract screening. The latter resulted in 73 articles eligible for full-text screening, of which only 39 met our inclusion criteria. Of note, 1 study was excluded due to the lack of variance/SD data in their reported outcomes. That being said, we found additional 3 articles by manual search, making the total number of included articles for quantitative synthesis as 42. We presented these steps in the PRISMA flow diagram in Figure 1.

### Characteristics of Included Studies

The baseline characteristics of included studies are summarized in Table 1. Overall, the sample size of included patients was 9027, ranging from 20 patients in 1 study^24^ to as high as 1359 patients in another study.^25^ According to the study design, 4 studies were prospective cohort, 4 were RCTs, and 34 were retrospective cohort studies. Other characteristics such as age, gender, country, and number of cases in the intervention (on-clamp) and control (off-clamp) groups are presented in Table 1.

### Quality Assessment

The quality of 38 nonrandomized studies (cohort studies) was assessed using the ROBINS-I ROB tool (Table 2), out of which 2 had an overall low ROB, 9 had serious ROB, and 27 had moderate ROB. On the other hand, in the 4 RCTs, 3 trials showed some concerns and 1 study showed low ROB with the use of the Cochrane revised ROB-II tool (2019) (Table 3).

## Study Endpoints

### Perioperative Outcomes

**Figure 2.**
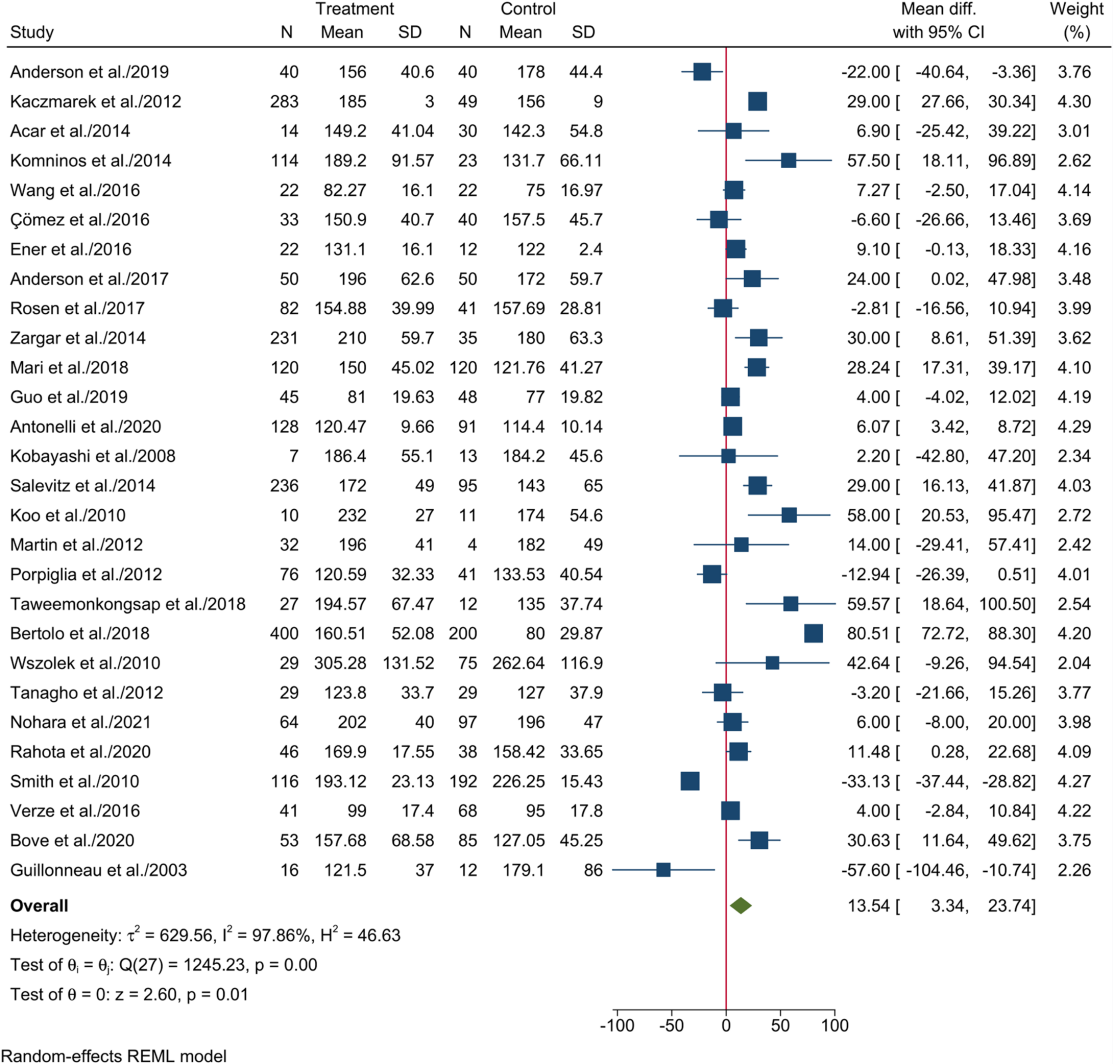
Forest plot showing the overall effect estimate of operative time between on-clamp and off-clamp procedures.

**Supplementary Figure 1.**
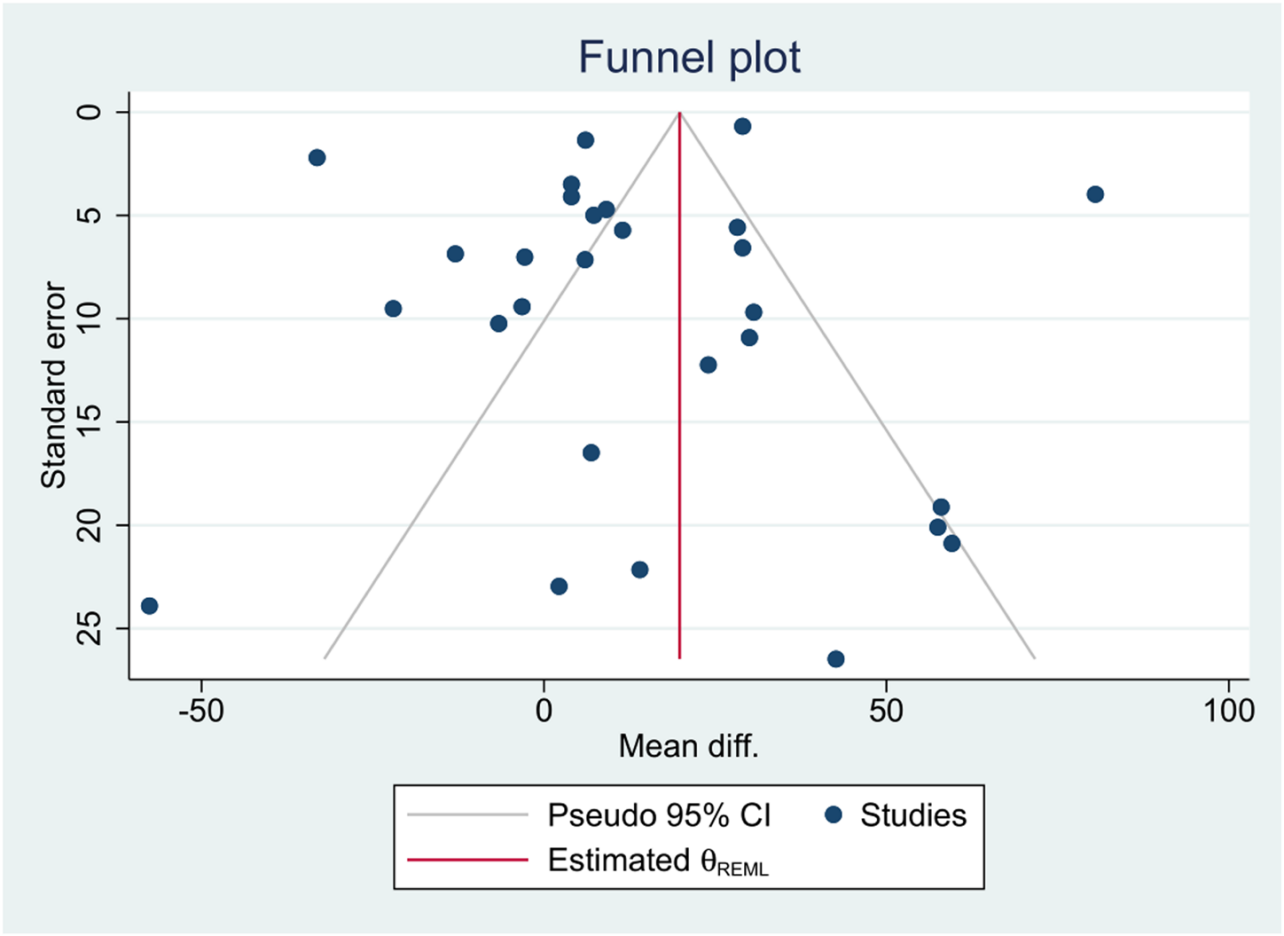
Funnel plot showing no risk of publication bias in terms of operative time

*Operative Time (Minutes)*: A total of 28 studies assessed operative time (Figure 2). Overall, on-clamp PN was associated with significantly higher operative time as compared to the off-clamp group [MD= 13.54 minutes; 95% CI: 3.34-23.74; *I* ^2^ = 97.86%]. In the light of significant heterogeneity, a meta-regression was performed based on sample size, and it revealed that sample size was a significant contributor to heterogeneity (*P* = .037). The leave-one-out sensitivity analysis did not reveal any significant change in the reported effect estimate. There was no risk of publication bias (Supplementary Figure 1).

**Figure 3.**
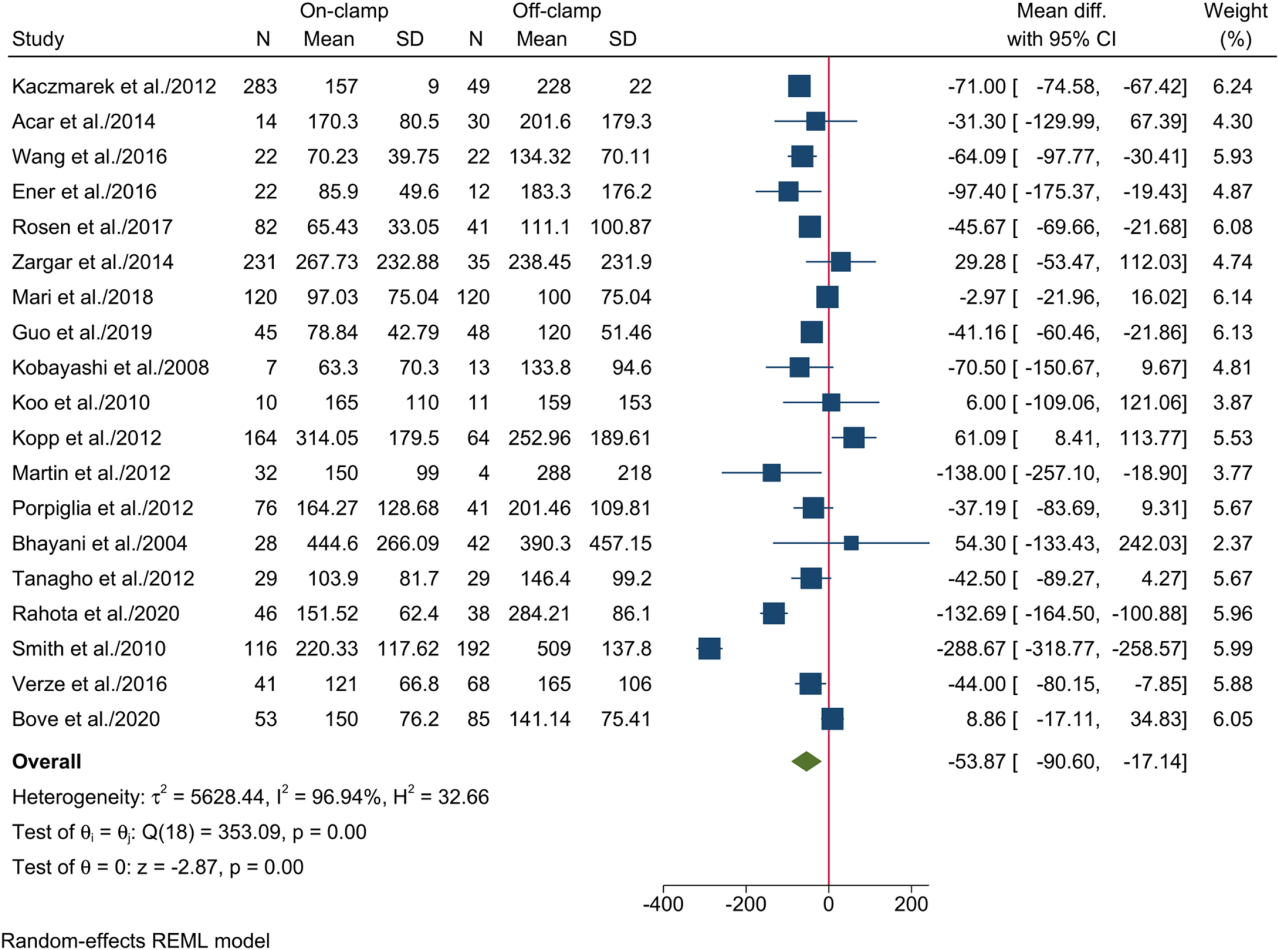
Forest plot showing the overall effect estimate of estimated blood loss between on-clamp and off-clamp procedures.

**Supplementary Figure 2.**
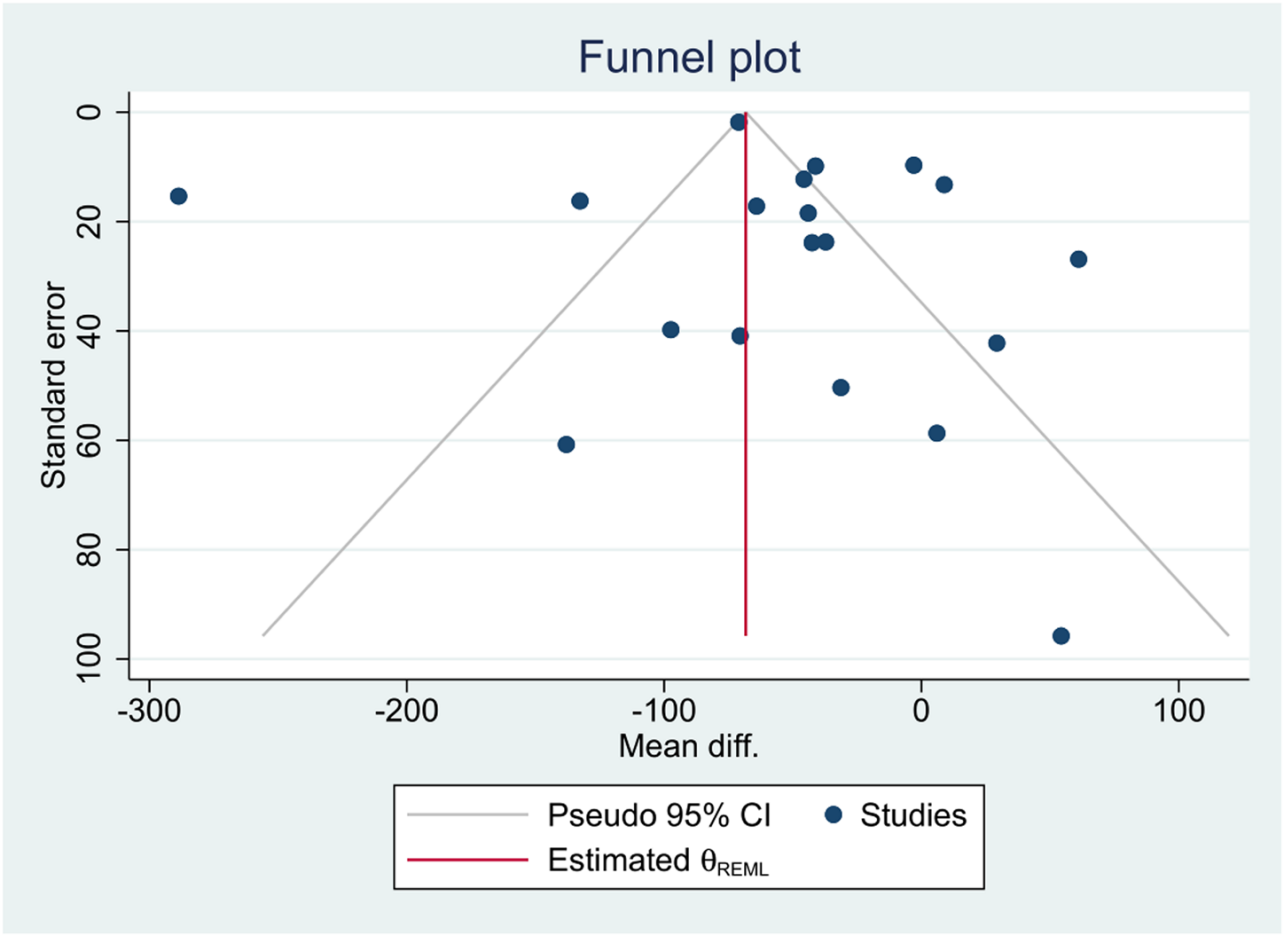
Funnel plot showing no risk of publication bias in terms of estimated blood loss

*Estimated Blood Loss (mL): *A total of 19 studies reported the estimated postoperative blood loss (Figure 3). Overall, the on-clamp technique was associated with significantly lower blood loss as compared to the off-clamp group [MD= –53.87 mL; 95% CI: –90.60–17.14; *I* ^2^ = 96.94%]. In the light of significant heterogeneity, a meta-regression was performed based on sample size; however, it was not a significant contributor to heterogeneity (*P* = .711). The leave-one-out sensitivity analysis did not reveal any significant change in the reported effect estimate. There was no risk of publication bias (Supplementary Figure 2).

**Figure 4.**
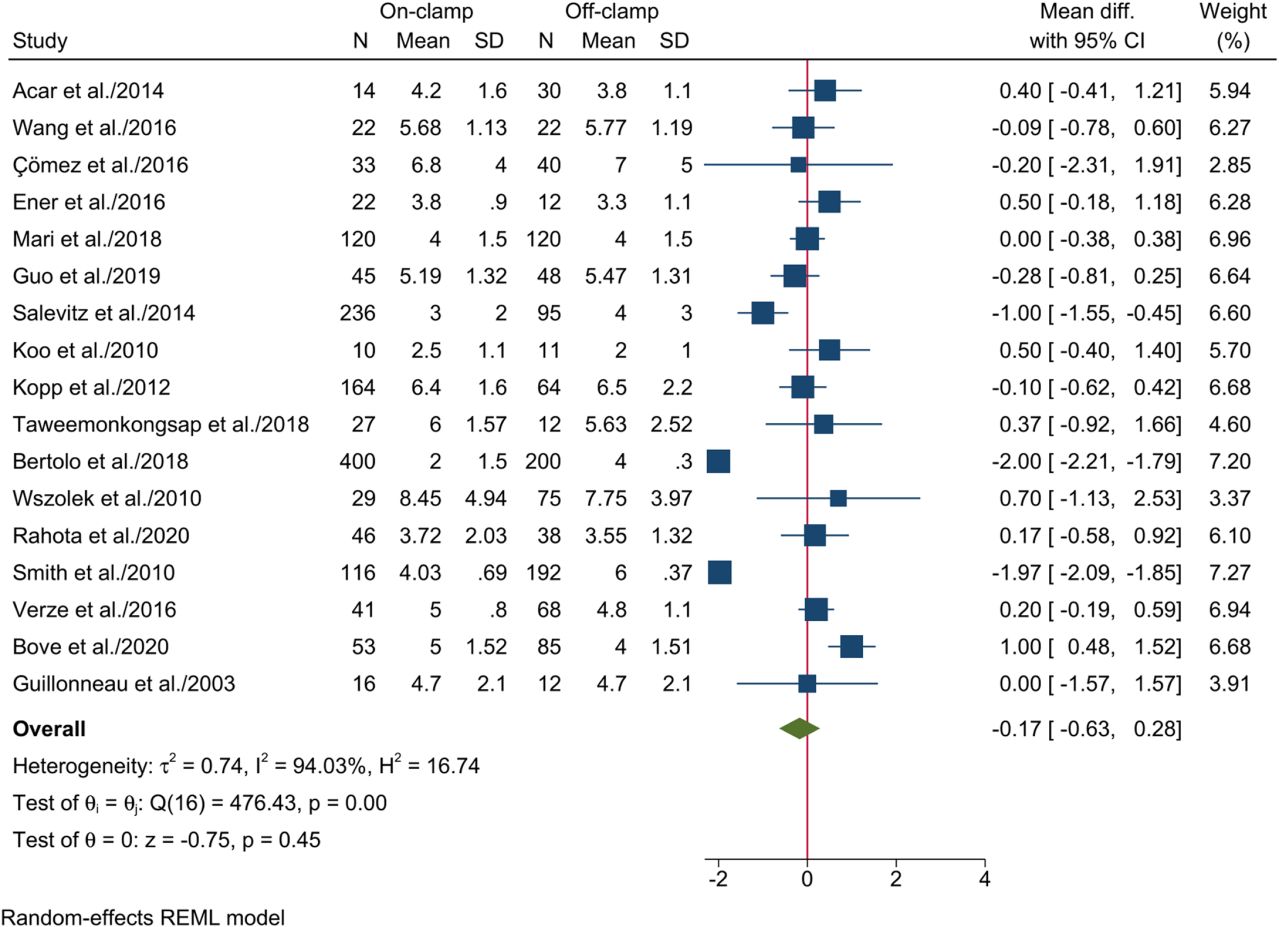
Forest plot showing the overall effect estimate of length of hospital stay between on-clamp and off-clamp procedures.

**Supplementary Figure 3.**
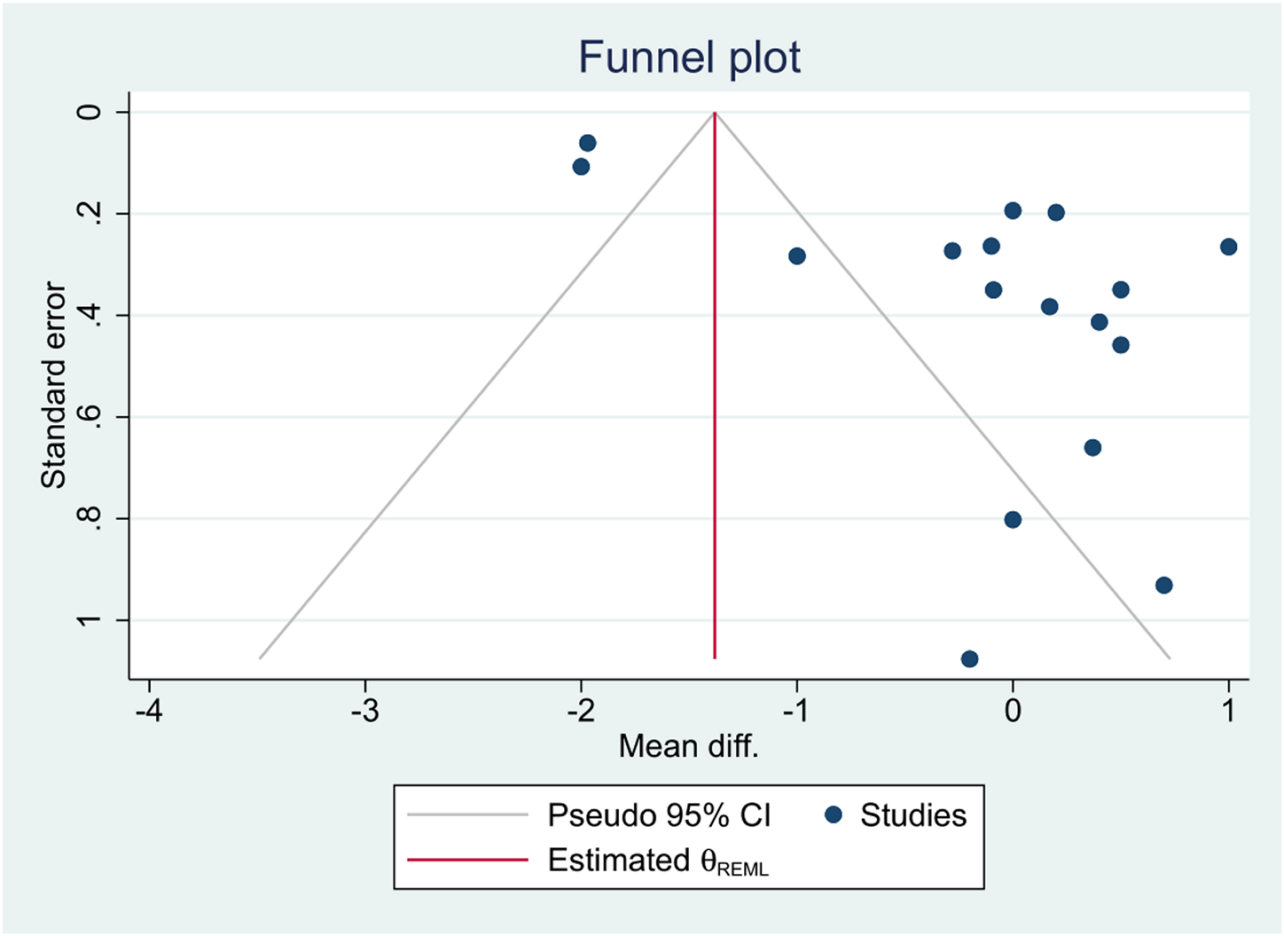
Funnel plot showing no risk of publication bias in terms of length of hospital stay

*Length of Hospital Stay (Days)*: A total of 17 studies reported the LoS (Figure 4). Overall, no significant difference was observed between the on-clamp and off-clamp techniques [MD= –0.17 days; 95% CI: –0.63: 0.28; *I* ^2^ = 94.03%]. In the light of significant heterogeneity, a meta-regression was performed based on sample size, and it was a highly significant contributor to heterogeneity (*P* = .001). The leave-one-out sensitivity analysis did not reveal any significant change in the reported effect estimate. There was no risk of publication bias (Supplementary Figure 3).

*Warm Ischemia Time (Minutes)*: A meta-analysis of this outcome was not feasible because, in the off-clamp group, the mean and SD of WIT was zero.

**Figure 5.**
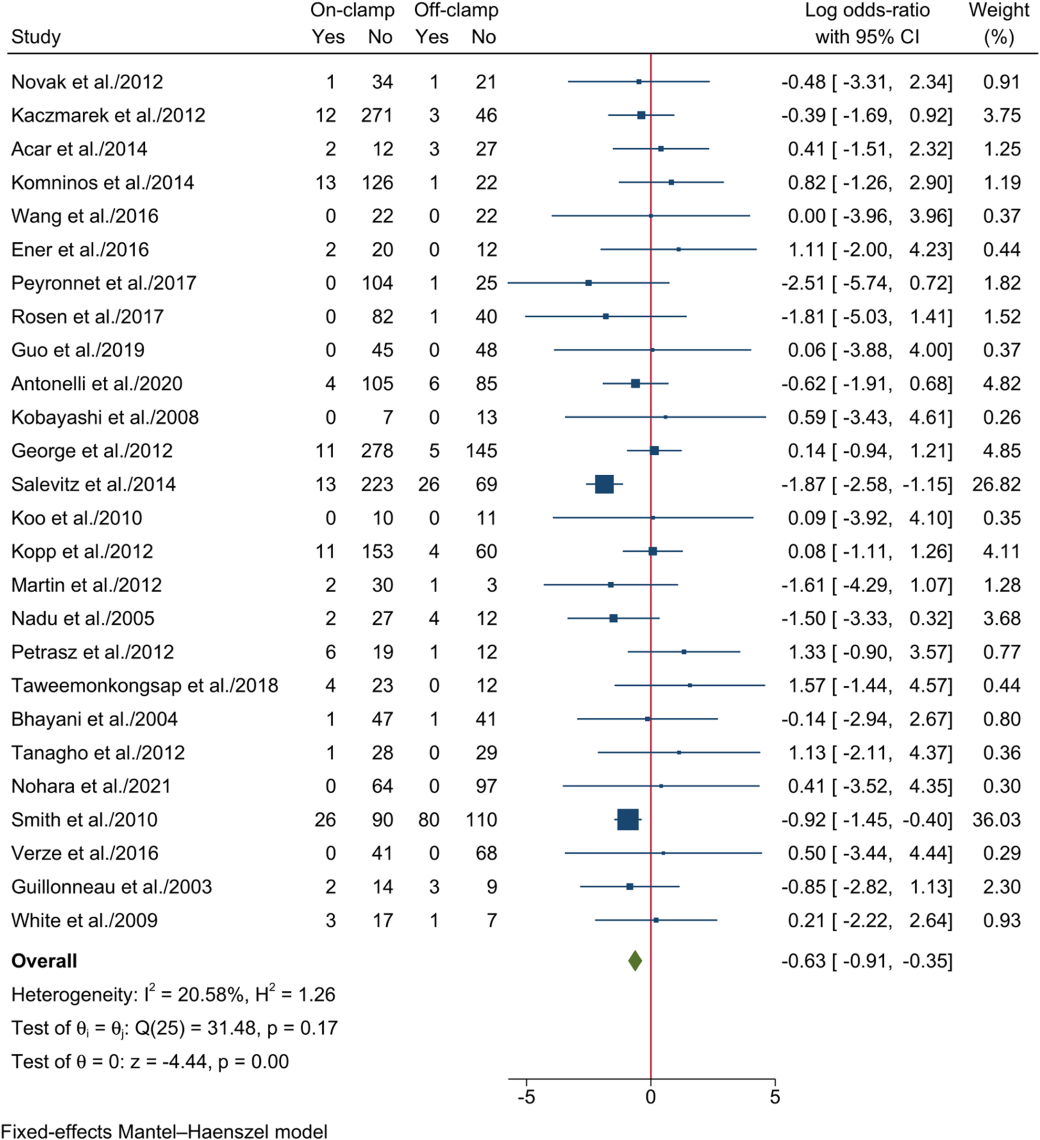
Forest plot showing the overall effect estimate of blood transfusion between on-clamp and off-clamp procedures.

**Supplementary Figure 4.**
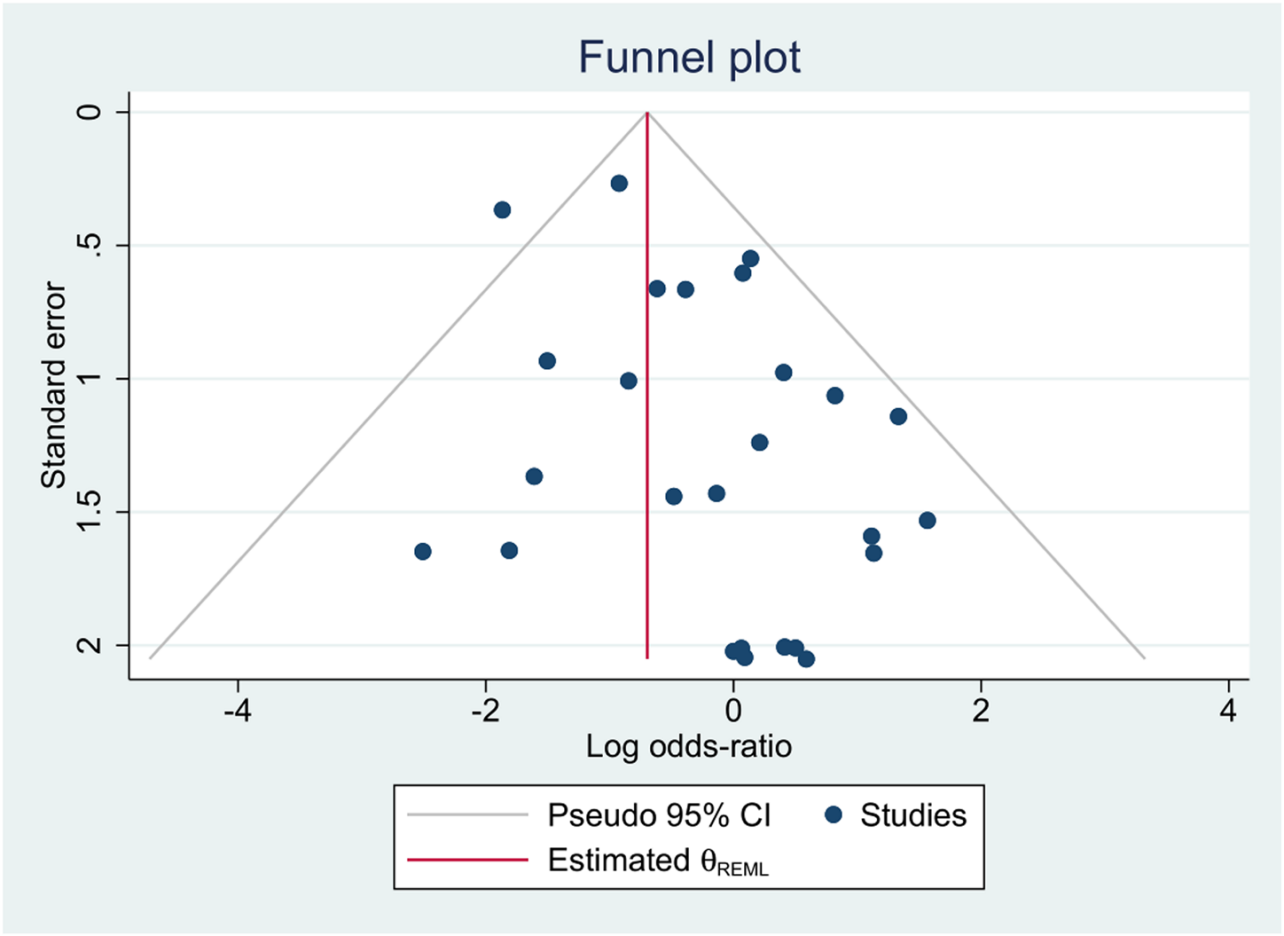
Funnel plot showing no risk of publication bias in terms of postoperative blood transfusion

*Postoperative blood transfusion: *A total of 26 studies reported postoperative blood transfusion (Figure 5). The on-clamp technique was associated with significantly lower risk for postoperative blood transfusion as compared to the off-clamp technique [logOR= –0.63; 95% CI: –0.91: –0.35; *I* ^2^ = 20.58%]. No significant heterogeneity was encountered, and the leave-one-out-sensitivity analysis did not reveal any significant change in the reported effect estimate. There was no risk of publication bias (Supplementary Figure 4).

## Safety Endpoints (Complications)

### Overall Complications

**Figure 6.**
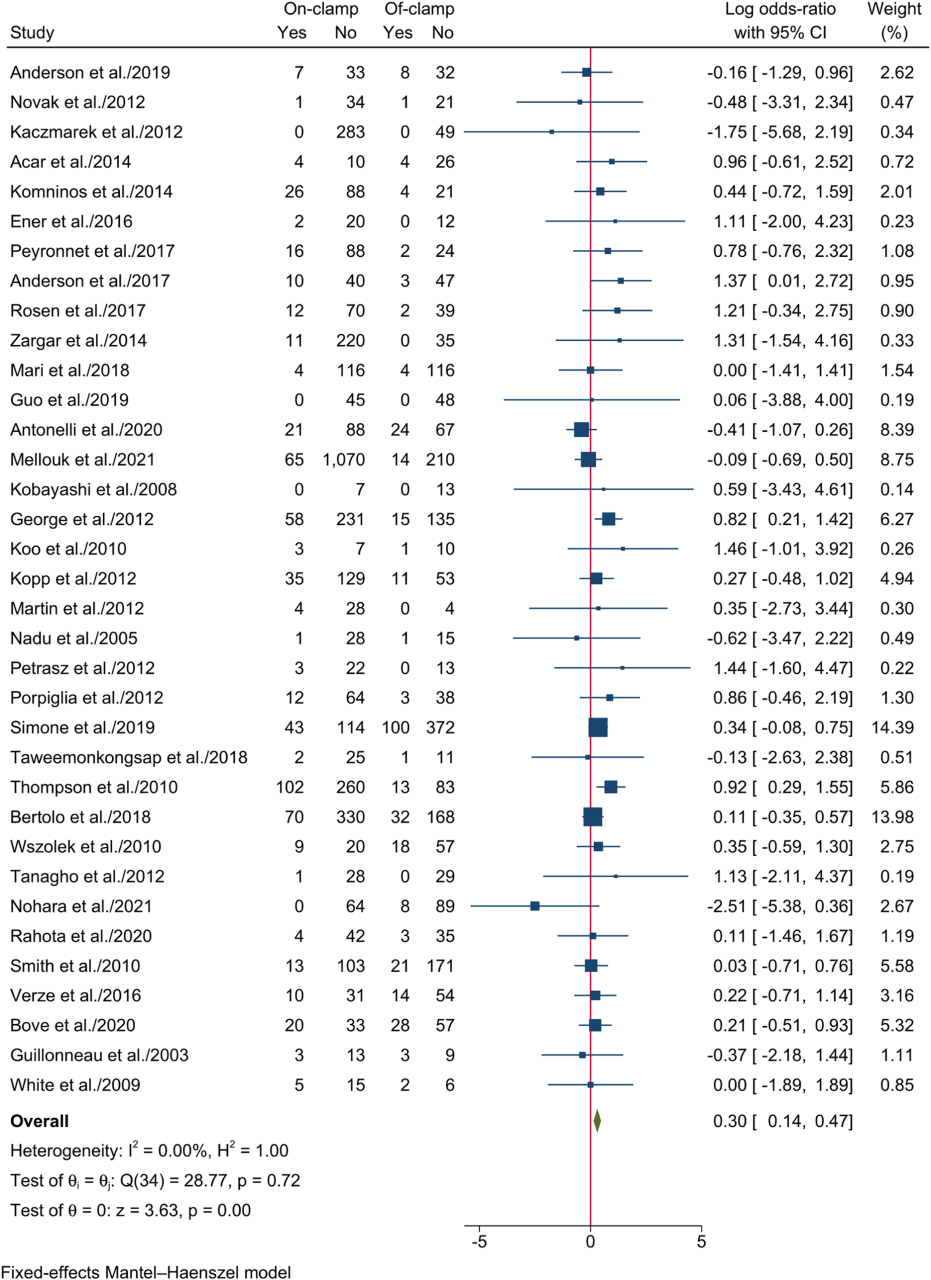
Forest plot showing the overall effect estimate of overall complications between on-clamp and off-clamp procedures.

**Supplementary Figure 5.**
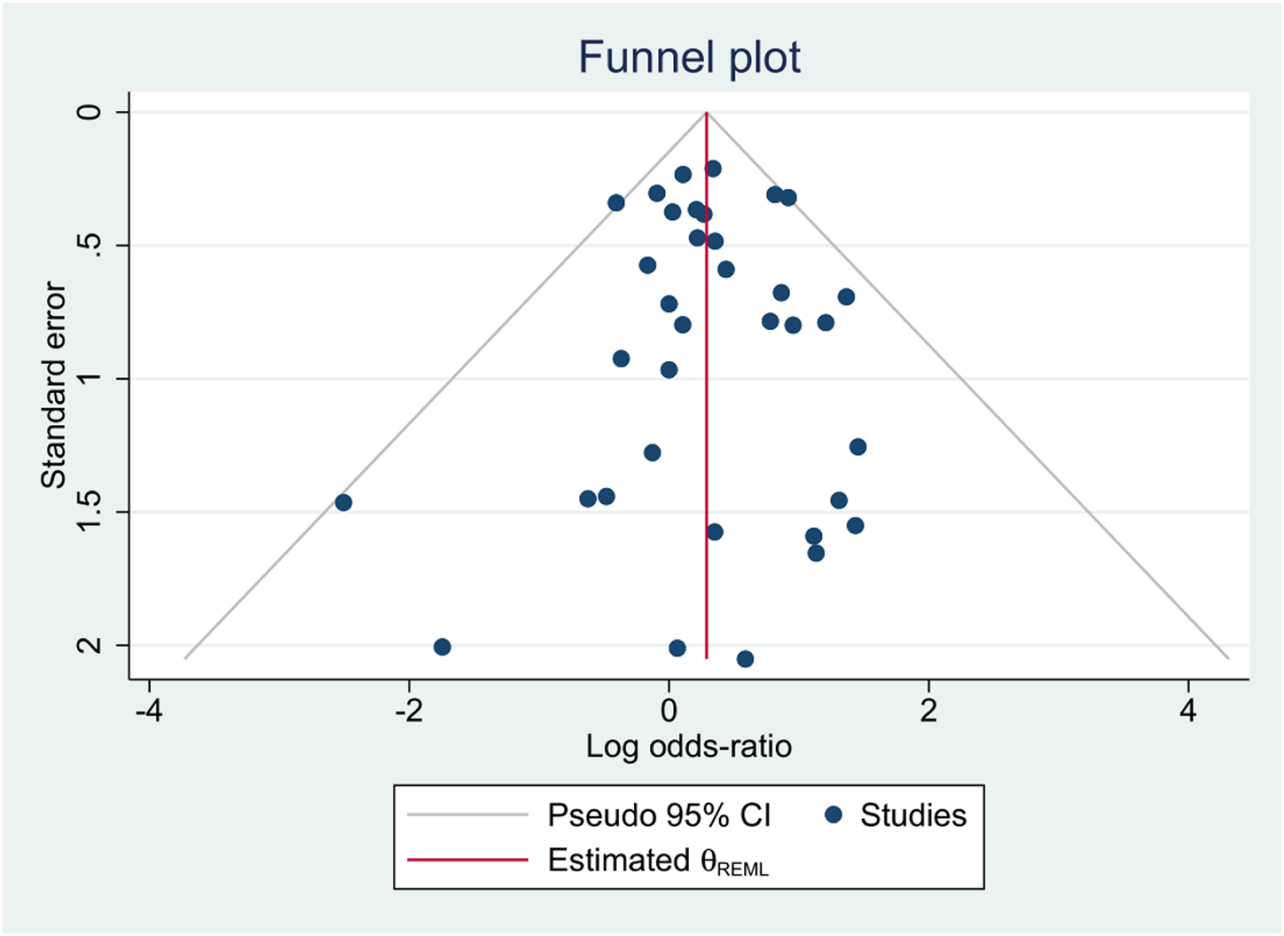
Funnel plot showing no risk of publication bias in terms of overall complications

A total of 35 studies reported postoperative complications following PN (Figure 6). Overall, the on-clamp technique was associated with significantly higher risk for postoperative complications as compared to the off-clamp technique [logOR= 0.30; 95% CI: 0.14: 0.47; *I* ^2^ = 0.00%]. No significant heterogeneity was encountered, and the leave-one-out-sensitivity analysis did not reveal any significant change in the reported effect estimate. There was no risk of publication bias (Supplementary Figure 5).

### Conversion to Open Surgery

**Figure 7.**
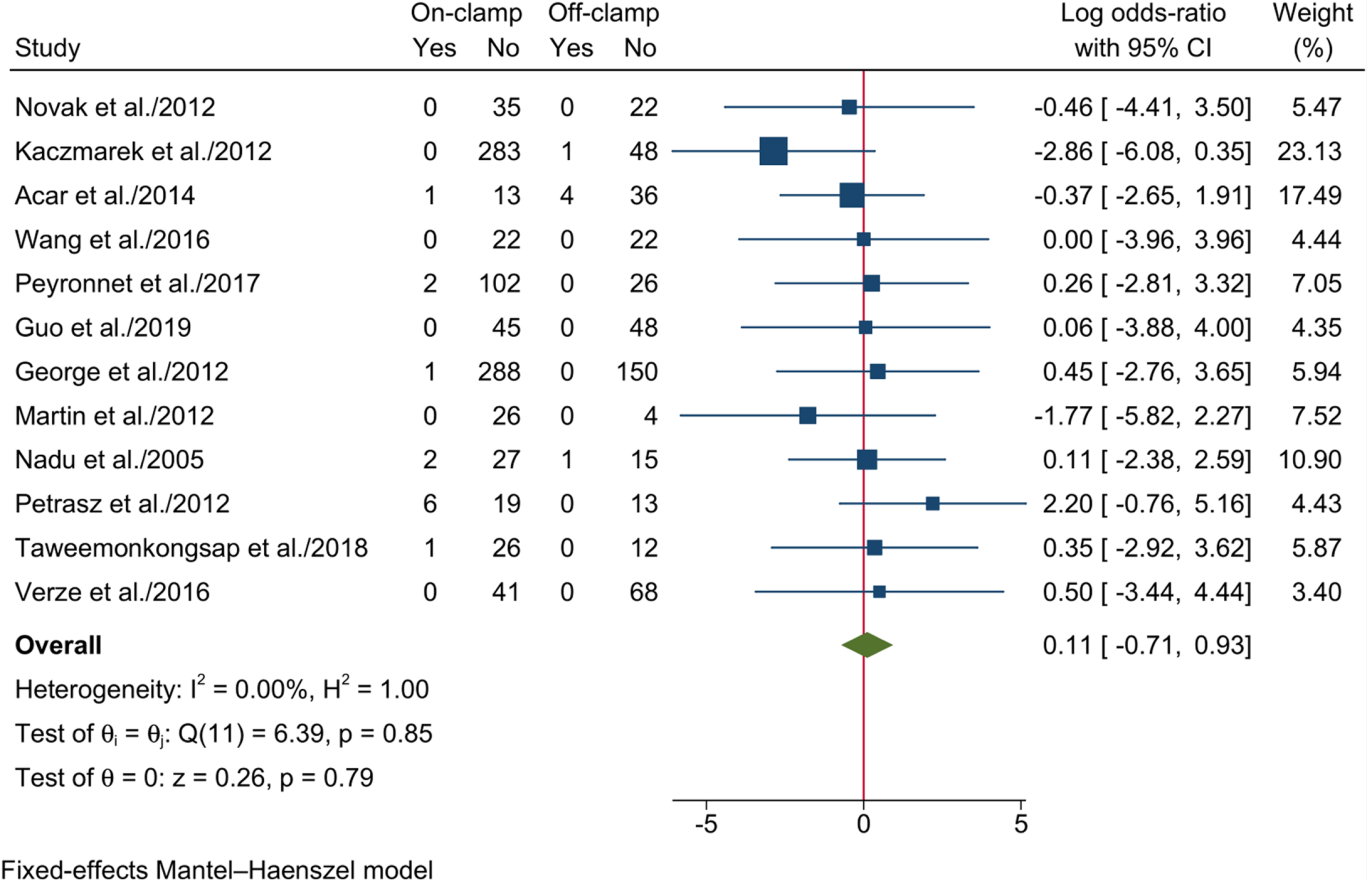
Forest plot showing the overall effect estimate of conversion to open surgery between on-clamp and off-clamp procedures.

**Supplementary Figure 6.**
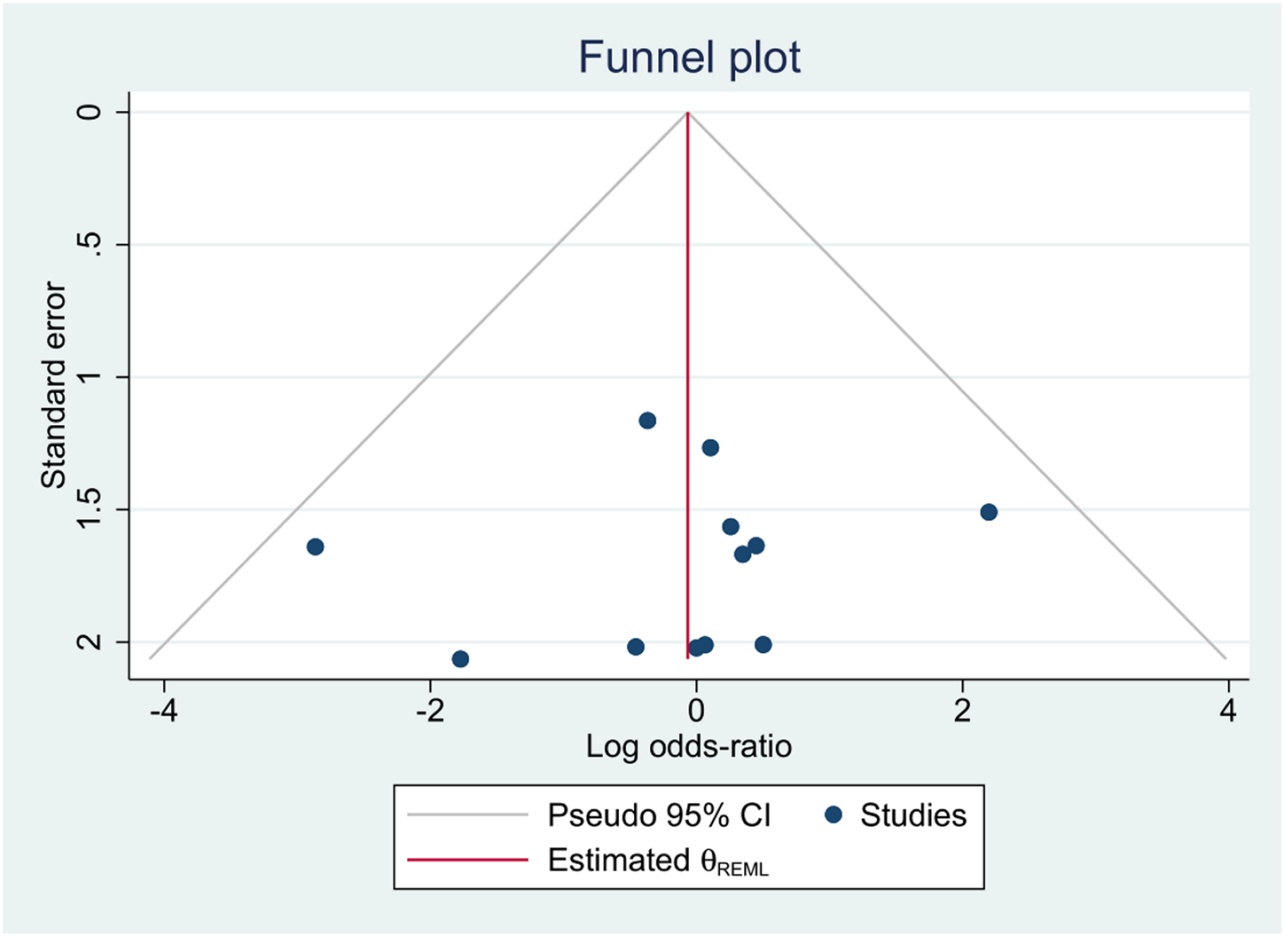
Funnel plot showing no risk of publication bias in terms of conversion to open surgery

A total of 12 studies reported surgical conversion to open surgery following PN (Figure 7). Overall, no significant difference was noted between on-clamp and off-clamp techniques [logOR= 0.11; 95% CI: –0.71: 0.93; *I* ^2^ = 0.00%]. No significant heterogeneity was encountered, and the leave-one-out-sensitivity analysis did not reveal any significant change in the reported effect estimate. There was no risk of publication bias (Supplementary Figure 6).

### Reintervention

A total of 6 studies reported reintervention following PN. Overall, no significant difference was noted between on-clamp and off-clamp techniques [logOR = 0.10; 95% CI: –0.89: 1.09; *I* ^2^ = 0.00%]. No significant heterogeneity was encountered, and the leave-one-out-sensitivity analysis did not reveal any significant change in the reported effect estimate.

### Major Bleeding

**Supplementary Figure 7.**
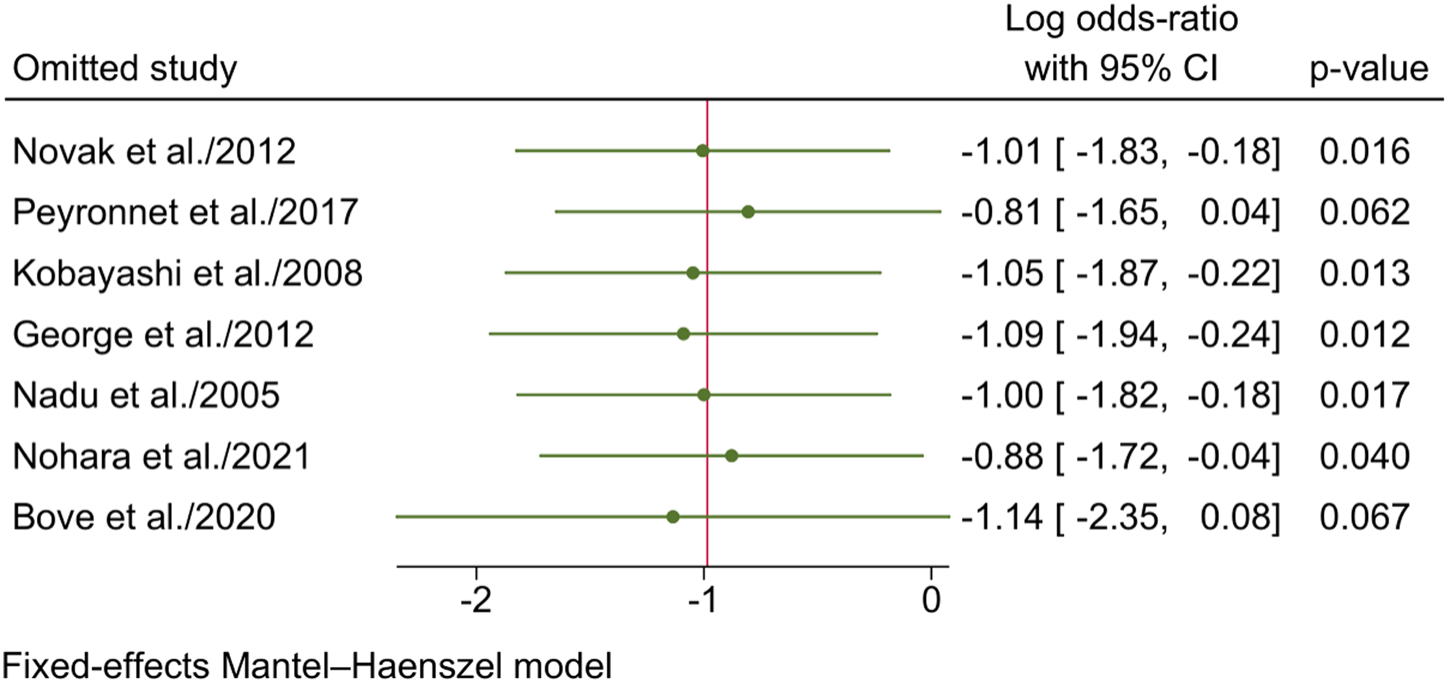
Leave-one-out sensitivity analysis revealing a significant change in the reported effect estimate of major bleeding

A total of 7 studies reported postoperative major bleeding following PN. Overall, the on-clamp technique was associated with lower risk for major bleeding when compared to the off-clamp method (logOR= –0.98; 95% CI: –1.79: –0.18; *I* ^2^ = 0.00%). No significant heterogeneity was encountered. Importantly, the leave-one-out-sensitivity analysis revealed a non-significant difference in major bleeding following the exclusion of the study of Peyronnet et al^26^ and Bove et al^27^ separately (Supplementary Figure 7).

### Any Bleeding

**Supplementary Figure 8.**
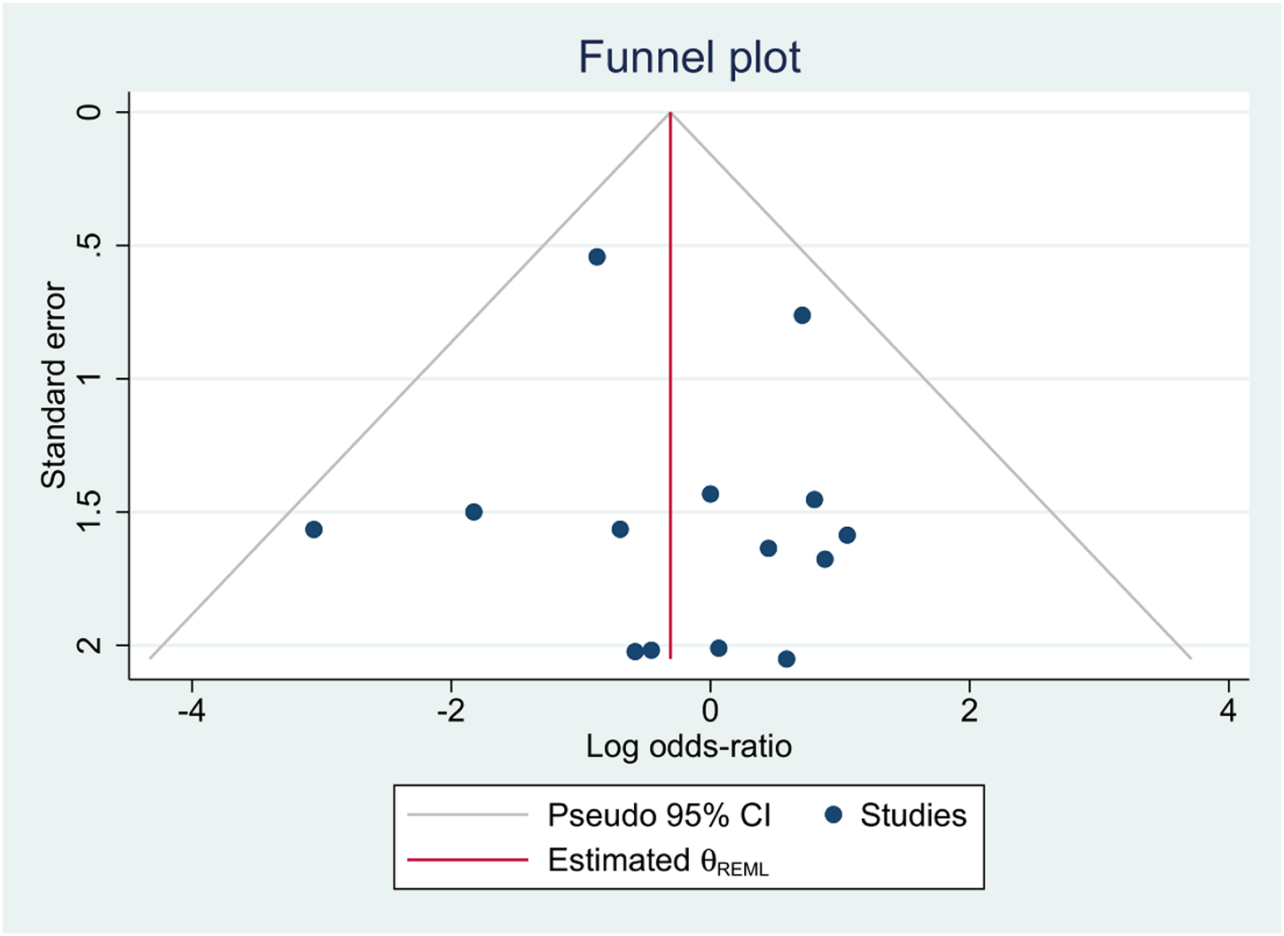
Funnel plot showing no risk of publication bias in terms of any bleeding

A total of 14 studies reported postoperative bleeding (any severity) following PN. Overall, no significant difference was noted between on-clamp and off-clamp techniques (logOR = –0.34; 95% CI: –0.90: 0.21; *I* ^2^ = 0.00%). No significant heterogeneity was encountered, and the leave-one-out-sensitivity analysis did not reveal any significant change in the reported effect estimate. No significant risk for publication bias was noted (Supplementary Figure 8).

### Acute Kidney Injury

**Supplementary Figure 9.**
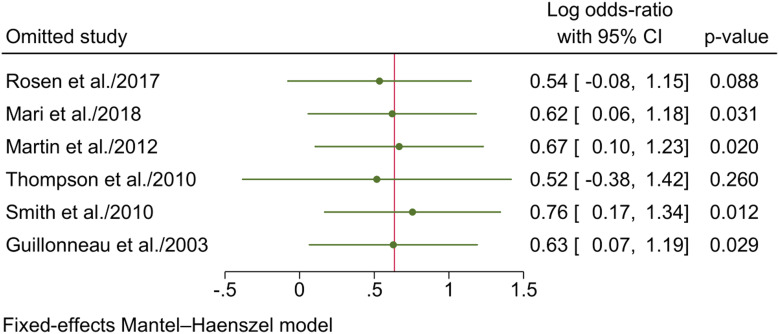
Leave-one-out sensitivity analysis revealing a significant change in the reported effect estimate of acute kidney injury

A total of 6 studies reported reintervention following PN. Overall, the on-clamp technique was associated with a significant increase in the risk of AKI as compared to the off-clamp technique (logOR = 0.63; 95% CI: 0.08: 1.19; *I* ^2^ = 0.00%). No significant heterogeneity was encountered. However, the leave-one-out sensitivity analysis revealed no significant difference between both groups following the exclusion of the study of Thompson et al^28^ (Supplementary Figure 9).

## Functional Outcomes

### Estimated Glomerular Filtration Rate

**Figure 8.**
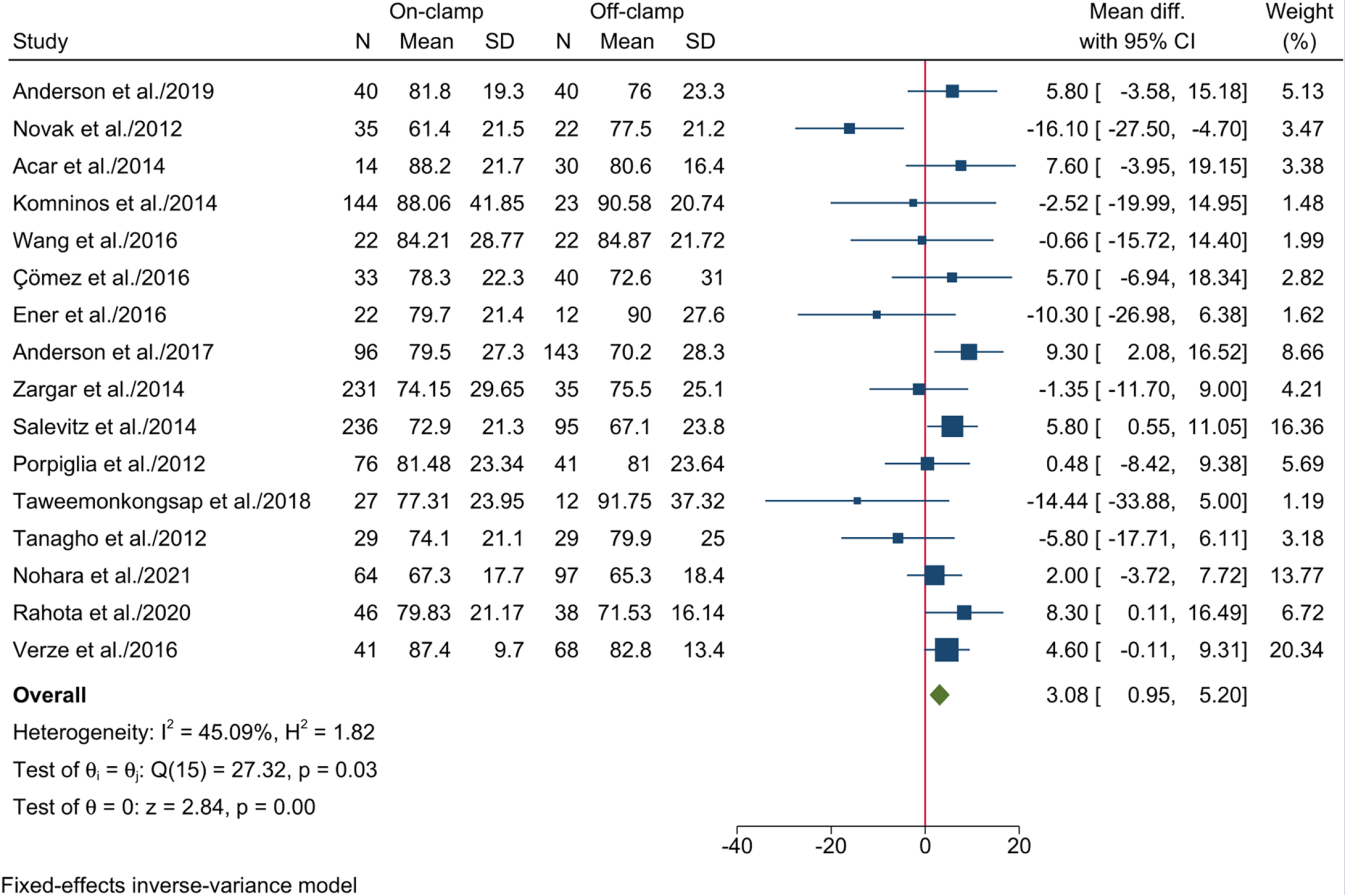
Forest plot showing the overall effect estimate of estimated glomerular filtration rate between on-clamp and off-clamp procedures.

**Supplementary Figure 10.**
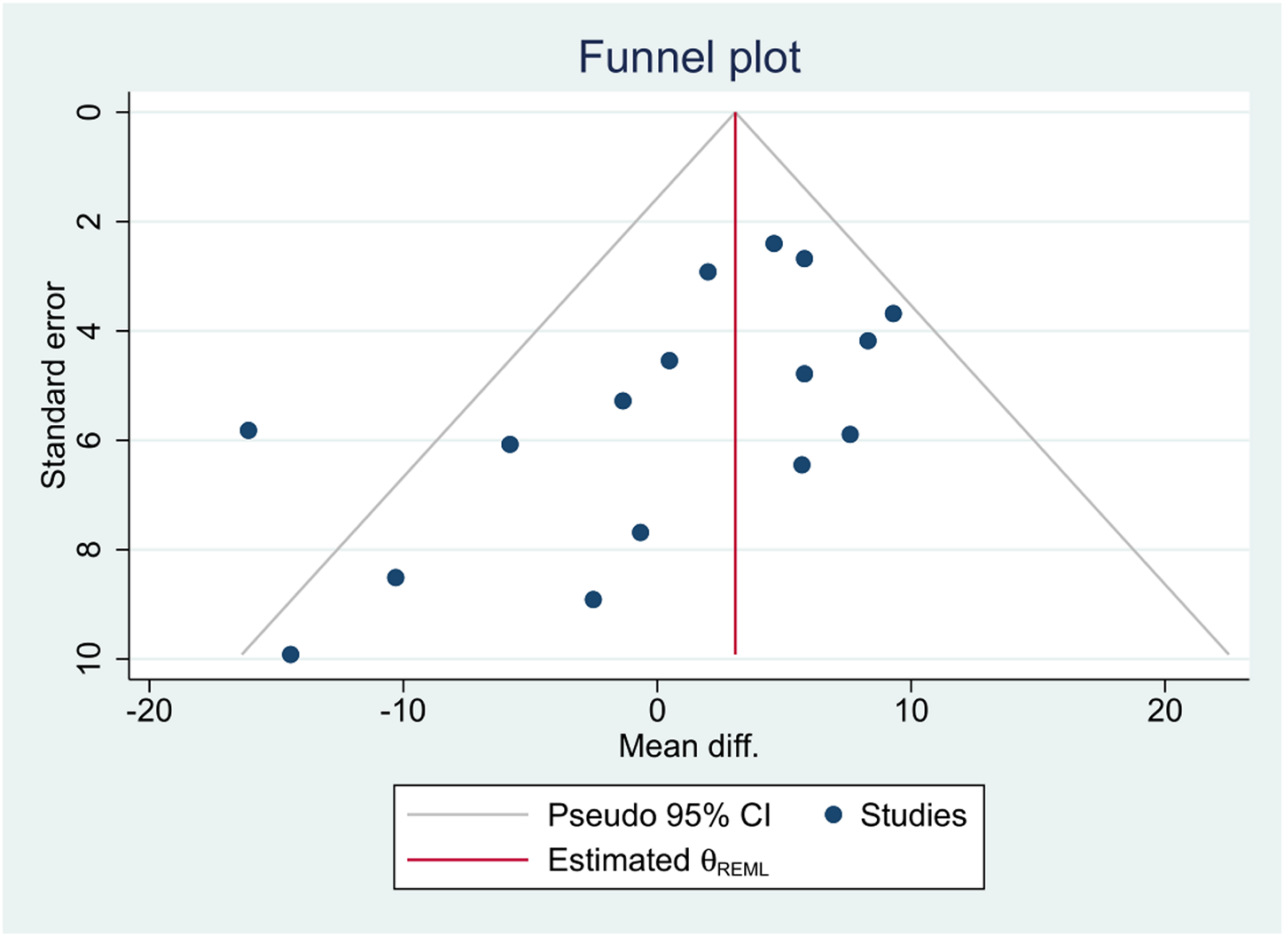
Funnel plot showing no risk of publication bias in terms of estimated glomerular filtration rate

A total of 16 studies reported the postoperative eGFR levels (Figure 8). Overall, the on-clamp technique was associated with significantly higher postoperative eGFR levels as compared to the off-clamp group (MD = 3.08; 95% CI: 0.95: 5.20: –17.14; *I* ^2^ =45.09%). No significant heterogeneity was encountered, and the leave-one-out sensitivity analysis did not reveal any significant change in the reported effect estimate. There was no significant risk of publication bias (Supplementary Figure 10).

### Percent Change in Estimated Glomerular Filtration Rate

A total of 4 studies reported the postoperative percent change in eGFR levels. Overall, no significant change was noted between the on-clamp and off-clamp methods (MD in percent change in eGFR= –1.75; 95% CI: –6.85: 3.34: –17.14; *I* ^2^ = 65.23%). In the light of significant heterogeneity, a meta-regression was performed, revealing no significant effect of sample size on the resultant heterogeneity (*P* = .853). The leave-one-out sensitivity analysis did not reveal any significant change in the reported effect estimate.

### Postoperative Hemoglobin

A total of 3 studies reported the postoperative Hb levels. Overall, no significant change was noted between the on-clamp and off-clamp methods (MD = 0.21; 95% CI: –1.14: 1.55; *I* ^2^ = 92.17%). In the light of significant heterogeneity, a meta-regression was performed revealing no significant effect of sample size on the resultant heterogeneity (*P* = .598). The leave-one-out sensitivity analysis did not reveal any significant change in the reported effect estimate.

### Postoperative Creatinine

A total of 7 studies reported the postoperative creatinine levels. Overall, no significant change was noted between the on-clamp and off-clamp methods (MD = 0.03; 95% CI: –0.07: 0.14; *I* ^2^ = 57.54%). In the light of significant heterogeneity, a meta-regression was performed revealing a significant contributing effect of sample size on the resultant heterogeneity (*P* = .003). The leave-one-out sensitivity analysis did not reveal any significant change in the reported effect estimate.

### Oncologic Outcomes

**Figure 9.**
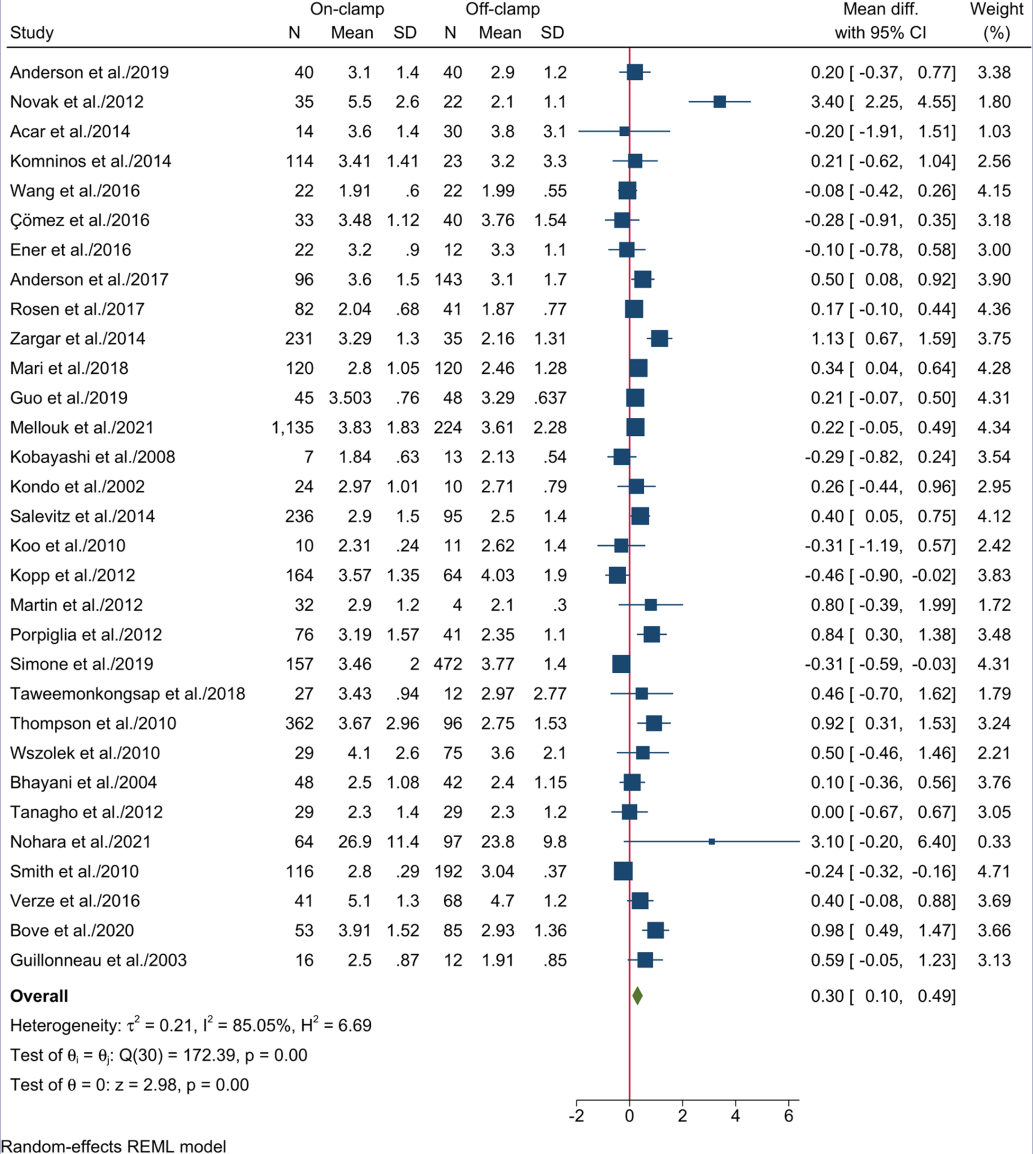
Forest plot showing the overall effect estimate of tumor size between on-clamp and off-clamp procedures.

**Supplementary Figure 11.**
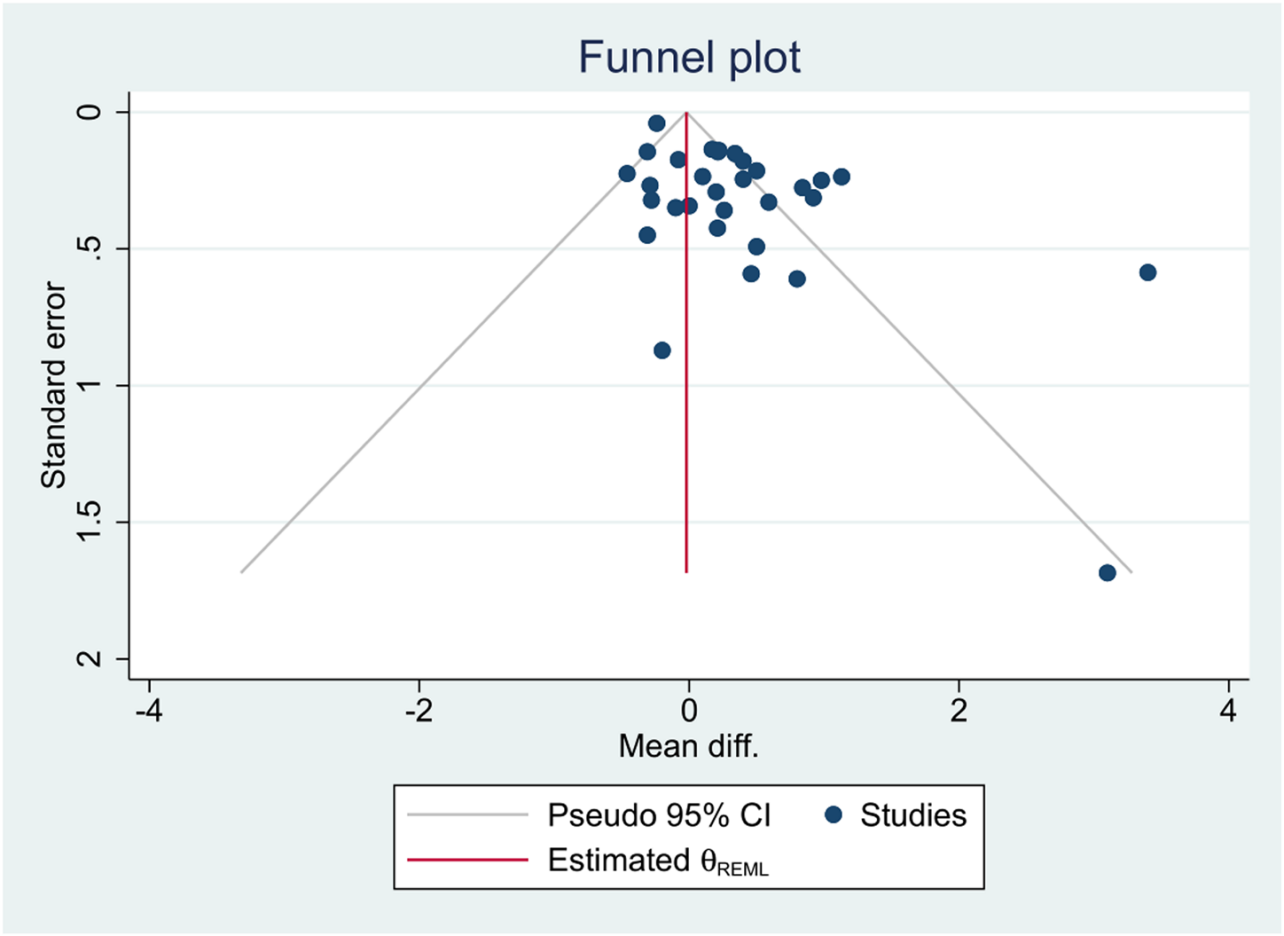
Funnel plot showing no risk of publication bias in terms of tumor size

*Tumor Size (cm): *A total of 31 studies reported tumor size (Figure 9). Overall, the on-clamp technique was associated with significantly higher tumor size as compared to the off-clamp group (MD= 0.30; 95% CI: 0.10: 0.49; *I* ^2^ = 85.05%). However, the clinical significance of this outcome should be carefully interpreted. In the light of significant heterogeneity, a meta-regression was performed revealing no significant effect of sample size on the resultant heterogeneity (*P* = .739). The leave-one-out sensitivity analysis did not reveal any significant change in the reported effect estimate, and no significant risk for publication bias was observed (Supplementary Figure 11).

**Supplementary Figure 12.**
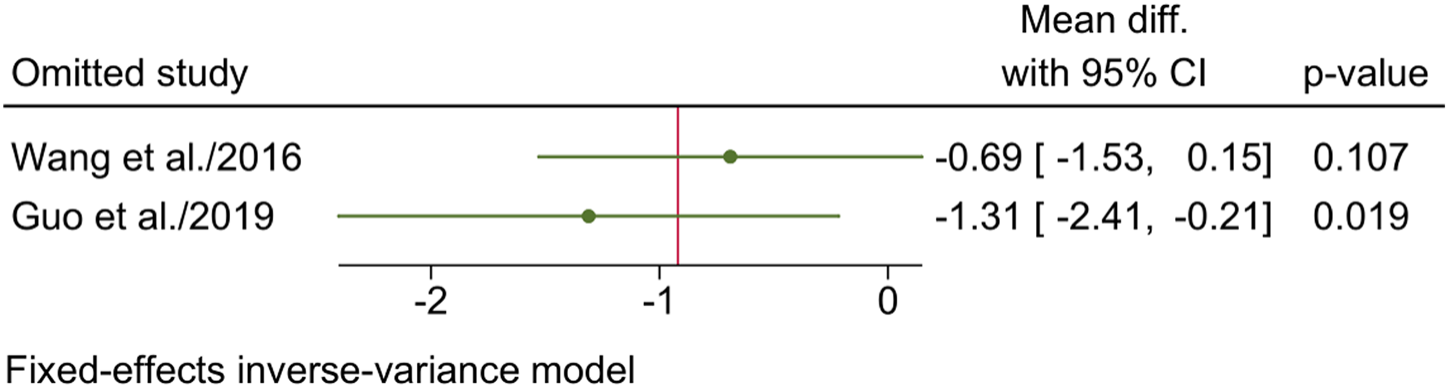
Leave-one-out sensitivity analysis revealing a significant change in the reported effect estimate of tumor resection time

*Tumor Resection Time (Minutes)*: Two studies reported the tumor resection time. Overall, the on-clamp technique was associated with significantly lower resection time as compared to the off-clamp group (MD= –0.92; 95% CI: –1.59: –0.25; *I* ^2^ = 0.00%). No significant heterogeneity was encountered, and the leave-one-out sensitivity analysis revealed no significant difference following the removal of 1 of both studies; however, this clinical applicability of this finding is negligent since the analysis was originally based on 2 studies (Supplementary Figure 12).

*Reconstruction Time (Minutes)*: Three studies reported the reconstruction time. Overall, no significant difference was noted between the on-clamp and off-clamp procedures (MD= 0.29; 95% CI: –1.97: 2.56; *I* ^2^ = 11.53%). No significant heterogeneity was encountered, and the leave-one-out sensitivity analysis revealed no significant difference in the reported effect estimate.

### Positive Surgical Margin

**Figure 10.**
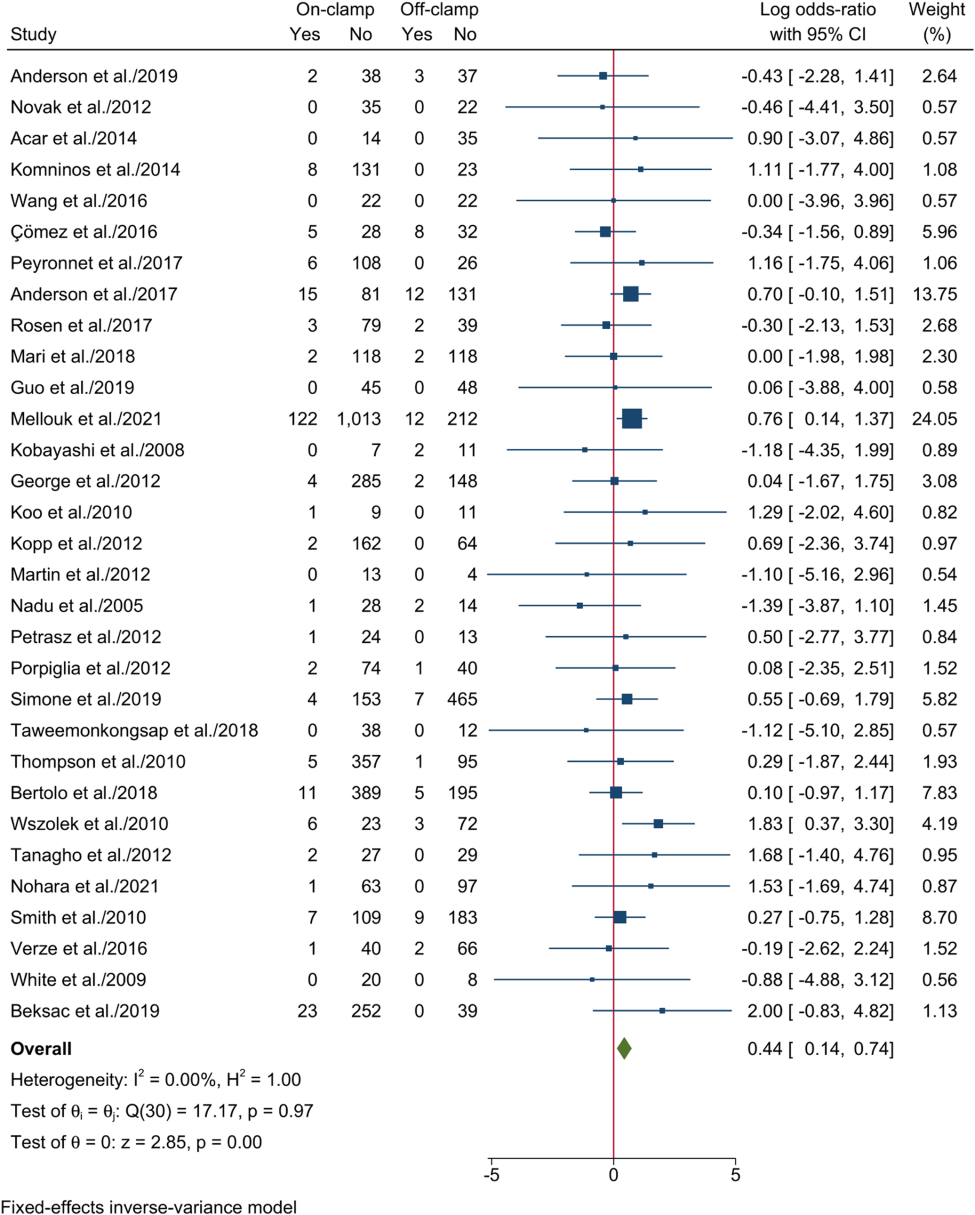
Forest plot showing the overall effect estimate of positive surgical margin between on-clamp and off-clamp procedures.

**Supplementary Figure 13.**
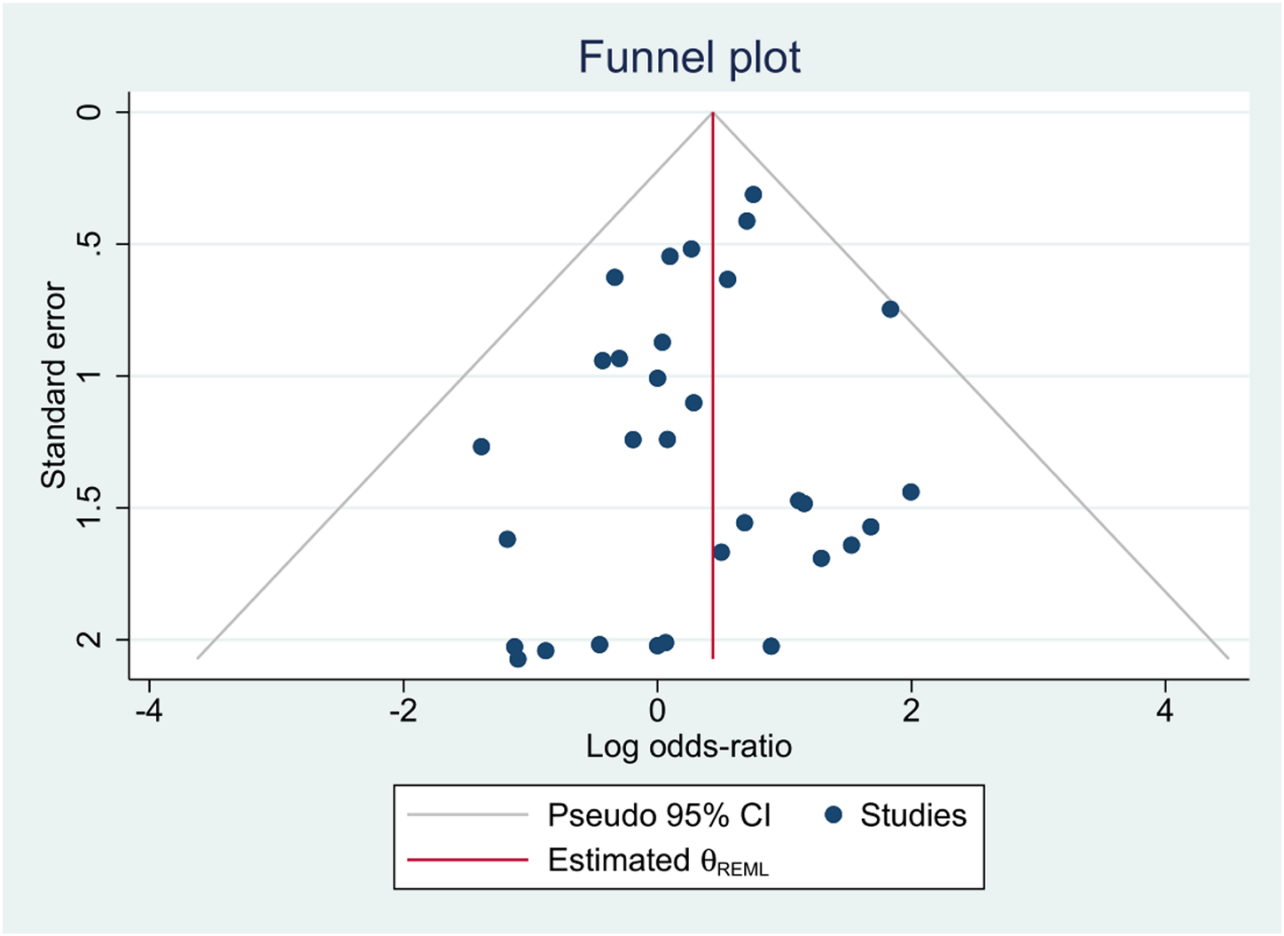
Funnel plot showing no risk of publication bias in terms of positivie surgical margin

A total of 31 studies assessed the postoperative positive surgical margin outcome (Figure 10). Overall, the on-clamp procedure was associated with significantly higher risk for postoperative positive surgical margin (logOR= 0.44; 95% CI: 0.14: 0.74; *I* ^2^ = 0.00%). No significant heterogeneity was encountered, and the leave-one-out sensitivity analysis revealed no significant change in the reported effect estimate. No significant risk of publication bias was observed (Supplementary Figure 13).

## Discussion

In the present systematic review, we aimed to provide the most comprehensive evidence comparing the safety and efficacy of off-clamp and on-clamp PN techniques. Accordingly, we have assessed various outcomes, including perioperative, functional, and oncologic outcomes. Regarding perioperative outcomes, we found that the operative time was significantly longer in the on-clamp versus off-clamp group. However, the results were significantly heterogeneous, and the sample size significantly contributed to the heterogeneity. Nevertheless, our findings are consistent with the results of the previous meta-analysis by Huang et al.^22^ The authors further reported that the overall complication rate was significantly higher in the on-clamp group, which is also consistent with our findings. However, these findings might be attributed to different factors, including the complexity of the tumor and technique, tumor size, the RENAL - Nephrometry Score between the 2 groups, and transition from off-clamp to on-clamp.^26,29,30^

Our findings also show that LoS and conversion rates did not differ significantly between the 2 groups, which is similar to the findings by Huang et al.^22^ We further found that EBL was significantly lower in the on-clamp group, which is consistent with Huang et al.^22^ These findings are logical because of the nature of the off-clamp technique. However, it should be noted that Huang et al^22^ demonstrated that these findings are clinically irrelevant because they found that transfusion rates were similar among the 2 groups. On the other hand, we found that the risk of postoperative blood transfusion was significantly lower in the on-clamp group, with no significant heterogeneity. However, we found no significant difference between both groups regarding any bleeding events. Moreover, sensitivity analysis showed that major bleeding events did not significantly differ between both groups. The reason behind current heterogeneous findings is unknown. However, it can be argued that the difference in characteristics of analyzed studies per each outcome might be the main reason for this heterogeneity. Otherwise, neither of the techniques should be considered superior to the other in this regard.

Regarding functional outcomes, we found that the on-clamp group had significantly higher postoperative eGFR levels. However, no significant difference was found between the 2 groups regarding postoperative percent change in eGFR levels. Furthermore, the meta-analyses by Huang et al^22^ and Cacciamani et al^30^ showed that postoperative renal function preservation was higher in the off-clamp group. On the other hand, the meta-analysis by Antonelli et al^21^ showed that eGFR variations did not significantly differ between the 2 groups on a long- and short-term basis. The heterogeneity among these findings might be attributed to the nature of included studies, as analysis of randomized clinical trials showed no difference.^33^ In contrast, data from cohort studies showed a functional advantage for the off-clamp approach.^34,35^ Our results also showed that postoperative creatinine and postoperative Hb did not significantly differ between the 2 groups. Moreover, sensitivity analysis showed that the risk of AKI did not significantly differ between the 2 groups. This might indicate the non-significant difference between the 2 techniques regarding renal outcomes.

Clamping, at the hilum, promotes precision in tumor resection in addition to the limitation of intraoperative bleeding; however, it causes a temporary interruption to the flow of blood which may cause ischemia that can subsequently lead to the deterioration of the renal function.^36^ Our analysis opposed this observation by highlighting a beneficial effect of the on-clamp technique on renal function (eGFR) as compared to the off-clamp method. This is contradictory to what has been reported in the literature, where a previous meta-analysis found that the off-clamp technique preserves renal function on the short- and long-terms, but with limited clinical significance (MD = 1.28; 95% CI: 0.04-2.48) and significant statistical heterogeneity (*P* = .04).^37^ Randomized trials in this regard found no significant difference between both techniques regarding renal function.^19,33^

Our finding is novel and still warrants further investigation for confirmation. However, our observation can be attributed to the wet ischemia time. For instance, a previous propensity score matching study indicated that higher WIT is predictive of renal functional deteriorations (defined as <30% reduction in eGFR postoperatively).^38^ The study found that WIT of >20 minutes was associated with increased risk of renal function deterioration by more than 2-folds (OR = 2.30; 95% CI: 1.13-4.64). On the other hand, WIT 20 minutes or less was not associated with renal function deterioration (*P* = .06). That being said, this observation cannot be confirmed in our meta-analysis due to the lack of sufficient and relevant data in this regard. Among studies included in the analysis, only 6 studies reported the mean WIT in both techniques (3 studies had a mean value >20 minutes^39-41^ and 3 studies had a mean value <20 minutes).^33,35,40^ But, no separate analyses based on WIT were provided, and thus, a meta-analysis could not be performed. Therefore, future studies should put this point into consideration which can provide insight into approaches that can be attempted to preserve patients’ renal function.

Regarding oncologic outcomes, we found that the on-clamp approach was associated with a significantly higher risk for postoperative positive surgical margin. On the other hand, no significant difference between the 2 groups was noticed regarding reconstruction time. Moreover, the on-clamp technique was associated with significantly lower resection time than the off-clamp group. However, the clinical applicability of this finding is negligent since the analysis was originally based on 2 studies. These findings are inconsistent with previous meta-analyses,^21,31^ which showed that positive surgical margin rates were comparable between the 2 groups. On the other hand, the meta-analysis by Huang et al^22^ showed that the positive surgical margin rate was significantly lower in the off-clamp group, indicating our findings. However, the authors further reported that both groups had similar recurrence rates. It is worth mentioning that we did not find a significant difference between the 2 groups regarding the rates of reinterventions. This might be due to the influence that might be caused on the tumor dissection technique by the clamping technique and/or tumor complexity, leading to different rates of positive surgical margins. Besides, we found that tumor size was significantly larger in the on-clamp group. A retrospective study by Shah et al^41^ showed that the risk of recurrence was significantly associated with positive surgical margins. However, this has been reported after PN. On the other hand, the association is still unclear after RAPN.

The current meta-analysis has some limitations that are worth mentioning. For instance, many current studies should be carefully interpreted due to the potential impact of confounders on the results. Moreover, most included studies were cohort studies, and only 4 were RCTs. Moreover, significant heterogeneity was observed in many of the analyzed outcomes. Although we conducted a sensitivity analysis and tried to reduce heterogeneity, some outcomes still had significant heterogeneity. Finally, the sample size of some included studies was relatively small (median: 113 patients). Therefore, we encourage future RCTs with proper sample sizes to be conducted for further validation of the current evidence.

## Conclusion

The current meta-analysis provides the most comprehensive comparison of the efficacy and safety between on-clamp and off-clamp PN approaches. Our findings indicate that most analyzed outcomes were comparable between the 2 groups. On the other hand, the superiority of the on-clamp technique was notable in EBL, risk of postoperative blood transfusion, postoperative eGFR levels, and tumor resection time. In contrast, the off-clamp technique was superior in other outcomes, including operative time, overall postoperative complications, risk of AKI, and postoperative post-surgical margin. However, current findings should be carefully interpreted due to some limitations that should be addressed in future more appropriate investigations.

## Figures and Tables

**Table 1.** Baseline Characteristics of Included Studies (n = 42)

Author/Year of Publication	Country	Sample	Design	On-clamp (n)	Off-clamp (n)	Age				Gender (Male)			
						On-clamp		Off-clamp		On-clamp		Off-clamp	
						Mean	SD	Mean	SD	Number	Total	Number	Total
Anderson et al/2019	USA	645.304 pt	RCT	40	40	59.4	11.2	56.6	9.8	29	40	22	40
Novak et al/2012	USA	57	Retrospective cohort	35	22	57	NR	55	NR	NR	NR	NR	NR
Kaczmarek et al/2012	USA	886	Retrospective cohort	283	49	60.2	0.5	60.4	1.5	161	283	25	49
Acar et al/2014	Turkey	40	Retrospective cohort	14	30	46.2	15.05	51.1	12.5	11	14	22	30
Komninos et al/2014	South Korea	180	Retrospective cohort	139	23	50.6	14.51	51.48	12.18	16	25	14	23
Wang et al/2016	China	44	Retrospective cohort	22	22	54.41	10.4	54.36	12.07	16	22	16	22
Çömez et al/2016	Turkey	73	Retrospective cohort	33	40	56.2	11.9	57.9	12.8	NR	NR	NR	NR
Ener et al/2016	Turkey	34	Retrospective cohort	22	12	54.4	10.1	53	8.2	NR	NR	NR	NR
Peyronnet et al/2017	France	130	Retrospective cohort	104	26	59.7	NR	63.8	NR	NR	NR	NR	NR
Anderson et al/2017	USA	100	Retrospective cohort	50	50	56.9	13.4	59.4	11.4	32	50	22	50
Rosen et al/2017	USA	123	Retrospective cohort	82	41	60.97	13.96	61.1	14.6	47	82	26	41
Zargar et al/2014	USA	266	Prospective cohort	231	35	58.15	11.7	57.6	14.7	150	231	22	35
Mari et al/2018	Italy	240	Prospective cohort	120	120	61.5	11.9	62.2	12.2	72	120	73	120
Guo et al/2019	China	93	Retrospective cohort	45	48	54.39	11.82	53.29	13.91	25	45	27	48
Antonelli et al/2020	Italy	302	RCT	150	152	62.3	12	63.9	12	88	150	92	152
Beksac et al/2019	USA	462	Prospective cohort	423	39	70	NR	69	NR	29	48	23	39
Antonelli et al/2022	Italy	324	RCT	160	164	62.92	3.01	65.76	3	86	160	99	164
Mellouk et al/2021	France	1359	Retrospective cohort	1135	224	60.33	12.81	61.04	12.74	725	1135	153	224
Kobayashi et al/2008	Japan	20	Retrospective cohort	7	13	51.8	10.7	60.3	13.5	6	7	10	13
George et al/2012	USA	439	Retrospective cohort	289	150	59.4	NR	59.2	NR	183	289	98	150
Kondo et al/2002	Japan	34	Retrospective cohort	24	10	53.9	12.5	54.5	15.1	NR	NR	NR	NR
Salevitz et al/2014	USA	331	Retrospective cohort	236	95	62	12	67	10	154	236	66	95
Koo et al/2010	USA	22	Retrospective cohort	10	11	63.7	12.4	58	15.1	NR	10	NR	11
Kopp et al/2012	USA	228	Retrospective cohort	164	64	57.4	14.7	55.9	15	96	164	44	64
Martin et al/2012	USA		Retrospective cohort	32	4	55	14	68	9	NR	NR	NR	NR
Nadu et al/2005	Israel	45	Retrospective cohort	29	16	67	NR	78	NR	NR	29	NR	16
Petrasz et al/2012	Poland	38	Retrospective cohort	25	13	58.3	NR	54.7	NR	NR	25	NR	13
Porpiglia et al/2012	Italy	117	Retrospective cohort	76	41	61.26	13.68	65.09	7.47	60	76	26	41
Simone et al/2019	Italy	629	Retrospective cohort	157	472	60.69	11.8	59.7	12	116	157	326	472
Taweemonkongsap et al/ 2018	Thailand	77	Retrospective cohort	65	12	60	11.3	50.1	14.9	28	38	6	12
Thompson et al/2010	USA	458	Retrospective cohort	362	96	61.72	12.65	62.37	10.27	246	362	74	96
Bertolo et al/2018	USA + Italy	600	Retrospective cohort	400	200	59.2	12.1	60.2	11.6	269	400	146	200
Wszolek et al/2010	USA	104	Retrospective cohort	29	75	57	12	63	11	20	29	41	75
Bhayani et al/2004	USA	118	Retrospective cohort	76	42	58	11.22	56.6	14.76	25	48	25	42
Tanagho et al/2012	USA	58	Retrospective cohort	29	29	60	13.4	60.8	12.1	NR	29	NR	29
Nohara et al/2021	Japan	161	Retrospective cohort	64	97	65.6	11.6	64.2	11.8	46	64	68	97
Rahota et al/2020	Romania	89	Retrospective cohort	46	38	57.2	12.8	58.8	12.04	30	46	26	38
Smith et al/2010	USA	306	Retrospective cohort	173	192	61.82	3.63	61.89	3.49	86	116	116	192
Verze et al/2016	Italy	109	Retrospective cohort	41	68	57.2	10.2	56.04	11.3	26	41	40	68
Bove et al/2020	Italy	138	RCT	53	85	60.59	12.19	60	10.56	37	53	63	85
Guillonneau et al/2003	France	28	Retrospective cohort	16	12	60	7.3	60.6	9.8	6	16	7	12
White et al/2009	USA	28	Prospective cohort	20	8	60.2	NR	59.3	NR	NR	20	NR	8

RCT, randomized controlled trial; NR, not reported.

**Table 2.** Risk of Bias Assessment of Nonrandomized Studies of Intervention Using the ROBINS-I tool

Author/Year of Publication	Confounding	Selection of Participants	Classification of Interventions	Deviation from Interventions	Missing Data	Measurement of Outcomes	Selection of Reported Results	Overall Bias
Novak et al/2012	Moderate	Low	Low	Low	Low	Low	Low	Moderate
Kaczmarek et al/2012	Moderate	Low	Low	Low	Low	Low	Low	Moderate
Acar et al/2014	Moderate	Low	Low	Low	Low	Low	Low	Moderate
Komninos et al/2014	Serious	Low	Low	Low	Low	Low	Low	Serious
Wang et al/2016	Serious	Low	Low	Low	Low	Low	Low	Serious
Çömez et al/2016	Low	Low	Low	Low	Low	Moderate	Low	Moderate
Ener et al/2016	Low	Low	Low	Low	Moderate	Moderate	Low	Moderate
Peyronnet et al/2017	Low	Low	Low	Low	Low	Moderate	Low	Moderate
Anderson et al/2017	Low	Low	Low	Low	Moderate	Moderate	Low	Moderate
Rosen et al/2017	Low	Low	Low	Low	Low	Moderate	Low	Moderate
Zargar et al/2014	Low	Low	Low	Low	Low	Moderate	Low	Moderate
Mari et al/2018	Serious	Low	Low	Low	Low	Moderate	Low	Serious
Guo et al/2019	Low	Low	Low	Low	Low	Low	Low	Low
Beksac et al/2019	Moderate	Low	Low	Low	Low	Low	Low	Moderate
Mellouk et al/2021	Serious	Low	Low	Low	Low	Low	Low	Serious
Kobayashi et al/2008	Serious	Low	Low	Low	Low	Low	Low	Serious
George et al/2012	Low	Low	Low	Low	Low	Moderate	Low	Moderate
Kondo et al/2002	Low	Low	Low	Low	Moderate	Moderate	Low	Moderate
Salevitz et al/2014	Low	Low	Low	Low	Low	Moderate	Low	Moderate
Koo et al/2010	Low	Low	Low	Low	Moderate	Moderate	Low	Moderate
Kopp et al/2012	Low	Low	Low	Low	Low	Moderate	Low	Moderate
Martin et al/2012	Low	Low	Low	Low	Low	Moderate	Low	Moderate
Nadu et al/2005	Serious	Low	Low	Low	Low	Moderate	Low	Serious
Petrasz et al/2012	Low	Low	Low	Low	Low	Low	Low	Low
Porpiglia et al/2012	Moderate	Low	Low	Low	Low	Low	Low	Moderate
Simone et al/2019	Moderate	Low	Low	Low	Low	Low	Low	Moderate
Taweemonkongsap et al/2018	Moderate	Low	Low	Low	Low	Low	Low	Moderate
Thompson et al/2010	Serious	Low	Low	Low	Low	Low	Low	Serious
Bertolo et al/2018	Serious	Low	Low	Low	Low	Low	Low	Serious
Wszolek et al/2010	Low	Low	Low	Low	Low	Moderate	Low	Moderate
Bhayani et al/2004	Low	Low	Low	Low	Moderate	Moderate	Low	Moderate
Tanagho et al/2012	Low	Low	Low	Low	Low	Moderate	Low	Moderate
Nohara et al/2021	Low	Low	Low	Low	Moderate	Moderate	Low	Moderate
Rahota et al/2020	Low	Low	Low	Low	Low	Moderate	Low	Moderate
Smith et al/2010	Low	Low	Low	Low	Low	Moderate	Low	Moderate
Verze et al/2016	Serious	Low	Low	Low	Low	Moderate	Low	Serious
Guillonneau et al/2003	Moderate	Low	Low	Low	Low	Low	Low	Moderate
White et al/2009	Moderate	Low	Low	Low	Low	Low	Low	Moderate

**Table 3.** Risk of Bias Assessment of Randomized Controlled Trials Using the Revised Cochrane ROB-II Tool (2019)

Author/Year of Publication	Randomization	Deviation from Intended Interventions	Missing Outcome Data	Outcome Measurement	Selective Reporting	Overall Bias
Anderson et al/2019	Low	Low	Low	Low	Low	Low
Antonelli et al/2020	Some concerns	Low	Low	Low	Some concerns	Some concerns
Antonelli et al/2022	Some concerns	Low	Low	Low	Low	Some concerns
Bove et al/2020	Low	Low	Low	Low	Some concerns	Some concerns

## References

[1] Agrawal S Sedlacek H Kim SP . Comparative effectiveness of surgical treatments for small renal masses. Urol Clin North Am. 2017;44(2):257 267. (10.1016/j.ucl.2016.12.011) 28411917

[2] Berg WT Tomaszewski JJ Yang H Corcoran A . Complications of renal surgery. Urol Clin North Am. 2017;44(2):275 288. (10.1016/j.ucl.2016.12.013) 28411919

[3] Kocher NJ Rjepaj C Lehman E Raman JD . Incidence and histologic ­features of mixed renal tumors. J Surg Oncol. 2018;117(3):430 433. (10.1002/jso.24879) 29044535

[4] Jonasch E Gao J Rathmell WK . Renal cell carcinoma. BMJ. 2014;349:g4797. (10.1136/bmj.g4797) PMC4707715 25385470

[5] Chow WH Dong LM Devesa SS . Epidemiology and risk factors for kidney cancer. Nat Rev Urol. 2010;7(5):245 257. (10.1038/nrurol.2010.46) 20448658 PMC3012455

[6] Herbert A Barclay ME Koo MM et al. Stage-specific incidence trends of renal cancers in the East of England, 1999-2016. Cancer Epidemiol. 2021;71(A):101883. (10.1016/j.canep.2020.101883) PMC7988458 33493782

[7] Campbell S Uzzo RG Allaf ME et al. Renal mass and localized renal cancer: AUA Guideline. J Urol. 2017;198(3):520 529. (10.1016/j.juro.2017.04.100) 28479239

[8] Motzer RJ Jonasch E Agarwal N et al. Kidney Cancer, version 2. 2017, NCCN Clinical Practice Guidelines in oncology. J Natl Compr Canc Netw. 2017;15(6):804 834. (10.6004/jnccn.2017.0100) 28596261

[9] Ljungberg B et al. European Association of Urology guidelines on renal cell carcinoma: the 2022 update. Eur Urol. 2022:82;e88. 35346519 10.1016/j.eururo.2022.03.006

[10] Scosyrev E Messing EM Sylvester R Campbell S Van Poppel H . Renal function after nephron-sparing surgery *versus* radical nephrectomy: results from EORTC randomized trial 30904. Eur Urol. 2014;65(2):372 377. (10.1016/j.eururo.2013.06.044) 23850254

[11] Ghani KR Sukumar S Sammon JD Rogers CG Trinh QD Menon M . Practice patterns and outcomes of open and minimally invasive partial nephrectomy since the introduction of robotic partial nephrectomy: results from the nationwide inpatient sample. J Urol. 2014;191(4):907 912. (10.1016/j.juro.2013.10.099) 24184365

[12] Shen Z Xie L Xie W et al. The comparison of perioperative outcomes of robot-assisted and open partial nephrectomy: a systematic review and meta-analysis. World J Surg Oncol. 2016;14(1):220. (10.1186/s12957-016-0971-9) PMC4994255 27549155

[13] Mari A Antonelli A Bertolo R et al. Predictive factors of overall and major postoperative complications after partial nephrectomy: results from a multicenter prospective study (The RECORd 1 project). Eur J Surg Oncol. 2017;43(4):823 830. (10.1016/j.ejso.2016.10.016) 27876194

[14] Schiavina R Mari A Antonelli A et al. A snapshot of nephron-sparing surgery in Italy: a prospective, multicenter report on clinical and ­perioperative outcomes (the RECORd 1 project). Eur J Surg Oncol. 2015;41(3):346 352. (10.1016/j.ejso.2014.12.001) 25583459

[15] Gill IS Eisenberg MS Aron M et al. “Zero ischemia” partial nephrectomy: novel laparoscopic and robotic technique. Eur Urol. 2011;59(1):128 134. (10.1016/j.eururo.2010.10.002) 20971550

[16] Mir MC Ercole C Takagi T et al. Decline in renal function after partial nephrectomy: etiology and prevention. J Urol. 2015;193(6):1889 1898. (10.1016/j.juro.2015.01.093) 25637858

[17] Antonelli A Cindolo L Sandri M et al. Safety of on- *vs* off-clamp robotic partial nephrectomy: per-protocol analysis from the data of the CLOCK randomized trial. World J Urol. 2020;38(5):1101 1108. (10.1007/s00345-019-02879-4) 31342246

[18] Anceschi U Brassetti A Bertolo R et al. On-clamp *versus* purely off-clamp robot-assisted partial nephrectomy in solitary kidneys: comparison of perioperative outcomes and chronic kidney disease progression at two high-volume centers. Minerva Urol Nephrol. 2021;73(6):739 745. (10.23736/S2724-6051.20.03795-9) 32573170

[19] Bertolo R Simone G Garisto J et al. Off-clamp *vs* on-clamp robotic partial nephrectomy: perioperative, functional and oncological outcomes from a propensity-score matching between two high-volume centers. Eur J Surg Oncol. 2019;45(7):1232 1237. (10.1016/j.ejso.2018.12.005) 30553632

[20] Mina-Riascos SH Vitagliano G García-Perdomo HA . Effectiveness and safety of partial nephrectomy-no ischemia *vs.* warm ischemia: systematic review and meta-analysis. Investig Clin Urol. 2020;61(5):464 474. (10.4111/icu.20190313) PMC7458877 32869563

[21] Antonelli A Veccia A Francavilla S et al. On-clamp *versus* off-clamp robotic partial nephrectomy: a systematic review and meta-analysis. Urologia. 2019;86(2):52 62. (10.1177/0391560319847847) 31179885

[22] Huang Y Cao D Chen Z et al. Comparison of perioperative, renal functional, and oncological outcomes between off-clamp and on-clamp robot-assisted partial nephrectomy for renal tumors: an updated evidence-based analysis. Front Oncol. 2021;11:730662. (10.3389/fonc.2021.730662) 34621676 PMC8490928

[23] Muka T Glisic M Milic J et al. A 24-step guide on how to design, conduct, and successfully publish a systematic review and meta-analysis in medical research. Eur J Epidemiol. 2020;35(1):49 60. (10.1007/s10654-019-00576-5) 31720912

[24] Kobayashi Y Saika T Manabe D Nasu Y Kumon H . The benefits of clamping the renal artery in laparoscopic partial nephrectomy. Acta Med Okayama. 2008;62(4):269 273. (10.18926/AMO/30939) 18766210

[25] Mellouki A Bentellis I Morrone A et al. Evaluation of oncological outcomes of robotic partial nephrectomy according to the type of hilar control approach (On-clamp *vs* Off-clamp), a multicentric study of the French network of research on kidney cancer-UROCCR 58-NCT03293563. World J Urol. 2021. (10.1007/s00345-020-03558-5) 33606044

[26] Peyronnet B Khene ZE Pradère B et al. Off-Clamp *versus* On-Clamp Robotic Partial nephrectomy: a Multicenter Match-Paired Case-Control Study. Urol Int. 2017;99(3):272 276. (10.1159/000471772) 28380483

[27] Bove P Bertolo R Sandri M et al. Deviation from the Protocol of a Randomized Clinical Trial Comparing On-Clamp *versus* Off-Clamp laparoscopic Partial nephrectomy (CLOCK II laparoscopic Study): a Real-Life Analysis. J Urol. 2021;205(3):678 685. (10.1097/JU.0000000000001417) 33035141

[28] Thompson RH Lane BR Lohse CM et al. Comparison of warm ischemia *versus* no ischemia during partial nephrectomy on a solitary kidney. Eur Urol. 2010;58(3):331 336. (10.1016/j.eururo.2010.05.048) 20557996

[29] Antonelli A Cindolo L Sandri M et al. Predictors of the transition from off to on clamp approach during ongoing robotic partial nephrectomy: data from the CLOCK randomized clinical trial. J Urol. 2019;202(1):62 68. (10.1097/JU.0000000000000194) 30827166

[30] Cacciamani GE Medina LG Gill TS et al. Impact of renal hilar control on outcomes of robotic partial nephrectomy: systematic review and cumulative meta-analysis. Eur Urol Focus. 2019;5(4):619 635. (10.1016/j.euf.2018.01.012) 29422419

[31] Anderson BG Potretzke AM Du K et al. Comparing off-clamp and on-clamp robot-assisted partial nephrectomy: a prospective randomized trial. Urology. 2019;126:102 109. (10.1016/j.urology.2018.11.053) 30659901

[32] Kaczmarek BF Tanagho YS Hillyer SP et al. Off-clamp robot-assisted partial nephrectomy preserves renal function: a multi-institutional propensity score analysis. Eur Urol. 2013;64(6):988 993. (10.1016/j.eururo.2012.10.009) 23122834

[33] Tanagho YS Bhayani SB Sandhu GS Vaughn NP Nepple KG Figenshau RS . Renal functional and perioperative outcomes of off-clamp *versus* clamped robot-assisted partial nephrectomy: matched cohort study. Urology. 2012;80(4):838 843. (10.1016/j.urology.2012.04.074) 22921704 PMC4142222

[34] Simone G Gill IS Mottrie A et al. Indications, techniques, outcomes, and limitations for minimally ischemic and off-clamp partial nephrectomy: a systematic review of the literature. Eur Urol. 2015;68(4):632 640. (10.1016/j.eururo.2015.04.020) 25922273

[35] Deng W et al. Off-clamp partial nephrectomy has a positive impact on short-and long-term renal function: a systematic review and meta-­analysis. BMC Nephrol. 2018;19(1):1 10. 30064370 10.1186/s12882-018-0993-3 PMC6069776

[36] Brassetti A Cacciamani GE Mari A et al. On-Clamp *vs.* off-Clamp Robot-Assisted Partial nephrectomy for cT2 Renal Tumors: retrospective Propensity-Score-Matched Multicenter Outcome Analysis. Cancers. 2022;14(18):4431. (10.3390/cancers14184431) PMC9496892 36139591

[37] Acar Ö Esen T Musaoğlu A Vural M . Do we need to clamp the renal hilum liberally during the initial phase of the learning curve of robot-assisted nephron-sparing surgery? Sci World J. 2014;2014:498917. (10.1155/2014/498917) PMC3944210 24688393

[38] Ener K Canda AE Altınova S et al. Impact of robotic partial nephrectomy with and without ischemia on renal functions: experience in 34 cases. Turk J Urol. 2016;42(4):272-277. (10.5152/tud.2016.67790) PMC5125742 27909621

[39] Komninos C Shin TY Tuliao P et al. Renal function is the same 6 months after robot‐assisted partial nephrectomy regardless of clamp technique: analysis of outcomes for off‐clamp, selective arterial clamp and main artery clamp techniques, with a minimum follow‐up of 1 year. BJU Int. 2015;115(6):921 928. (10.1111/bju.12975) 25376793

[40] Verze P Fedelini P Chiancone F et al. Perioperative and renal functional outcomes of laparoscopic partial nephrectomy (LPN) for renal tumours of high surgical complexity: a single-institute comparison between clampless and clamped procedures. World J Urol. 2017;35(3):403 409. (10.1007/s00345-016-1882-7) 27324881

[41] Shah PH Moreira DM Okhunov Z et al. Positive surgical margins increase risk of recurrence after partial nephrectomy for high risk renal tumors. J Urol. 2016;196(2):327 334. (10.1016/j.juro.2016.02.075) 26907508 PMC9235535

